# Disarming *Staphylococcus aureus*: Review of Strategies Combating This Resilient Pathogen by Targeting Its Virulence

**DOI:** 10.3390/pathogens14040386

**Published:** 2025-04-15

**Authors:** Abdelaziz Touati, Nasir Adam Ibrahim, Takfarinas Idres

**Affiliations:** 1 Laboratory of Microbial Ecology, FSNV, University of Bejaia, Bejaia 06000, Algeria; 2 Department of Biology, College of Science, Imam Mohammad Ibn Saud Islamic University (IMSIU), Riyadh 13318, Saudi Arabia; naabdalneim@imamu.edu.sa; 3 Laboratory for Livestock Animal Production and Health Research, Rabie Bouchama National Veterinary School of Algiers, Issad ABBAS Street, BP 161 Oued Smar, Algiers 16059, Algeria; t.idres@ensv.dz

**Keywords:** *Staphylococcus aureus*, anti-virulence strategies, adhesion and biofilm inhibition, toxin neutralization, QS inhibition

## Abstract

*Staphylococcus aureus* is a formidable pathogen notorious for its antibiotic resistance and diverse virulence mechanisms, including toxin production, biofilm formation, and immune evasion. This article explores innovative anti-virulence strategies to disarm *S. aureus* by targeting critical virulence factors without exerting bactericidal pressure. Key approaches include inhibiting adhesion and biofilm formation, neutralizing toxins, disrupting quorum sensing (e.g., Agr system inhibitors), and blocking iron acquisition pathways. Additionally, interventions targeting two-component regulatory systems are highlighted. While promising, challenges such as strain variability, biofilm resilience, pharmacokinetic limitations, and resistance evolution underscore the need for combination therapies and advanced formulations. Integrating anti-virulence strategies with traditional antibiotics and host-directed therapies offers a sustainable solution to combat multidrug-resistant *S. aureus*, particularly methicillin-resistant strains (MRSA), and mitigate the global public health crisis.

## 1. Introduction

*Staphylococcus aureus* is a formidable pathogen due to its diverse virulence arsenal, enabling it to cause infections ranging from superficial skin lesions to life-threatening systemic diseases [1]. Cytolytic toxins, such as pore-forming proteins and superantigens, are central to its pathogenicity, disrupting host cell membranes and provoking dysregulated immune responses. Exfoliative toxins and epidermal cell differentiation inhibitors also contribute to tissue damage, facilitating bacterial dissemination. The pathogen’s ability to form biofilms enhances its persistence on medical devices and host tissues, while immune evasion strategies—including protein A-mediated antibody neutralization and complement inhibition—thwart host defenses [1,2,3].

Virulence regulation is orchestrated by intricate systems such as the accessory gene regulator (Agr) quorum sensing (QS) network, which synchronizes toxin production and biofilm dynamics in response to population density [4,5]. However, functional redundancy among virulence factors, such as overlapping toxin activities and alternative regulatory pathways (e.g., two-component systems, SarA family proteins), complicates therapeutic targeting. Strain-specific variations further exacerbate this challenge, as dominant lineages exhibit distinct virulence profiles, influenced by mobile genetic elements like pathogenicity islands and phages. These elements facilitate horizontal gene transfer, driving the evolution of hypervirulent clones such as methicillin-resistant *S. aureus* (MRSA). Despite advances in understanding virulence regulation, translating this knowledge into therapies remains hindered by the pathogen’s adaptability and the dynamic host–pathogen interface [1,4,6,7].

The rise of multidrug-resistant *S. aureus*, particularly MRSA and vancomycin-resistant strains (VRSA), presents a critical public health crisis [8]. Resistance is primarily mediated by genetic elements such as staphylococcal chromosomal cassette *mec* (SCC*mec*), which harbors *mecA* or *mecC* genes encoding penicillin-binding protein 2a (PBP2a), conferring beta-lactam resistance [9]. Horizontal gene transfer via plasmids, transposons, and bacteriophages disseminates resistance determinants across strains, while biofilm formation and efflux pump overexpression further shield bacteria from antimicrobials [10].

MRSA’s epidemiological success is evident in its classification as an ESKAPE pathogen, with epidemic clones evolving through antibiotic selection pressure and genetic diversification [11]. Resistance extends beyond beta-lactams, including glycopeptides, fluoroquinolones, and oxazolidinones, limiting therapeutic options. Even newer agents, such as ceftaroline and tedizolid, face emerging resistance, highlighting the limitations of traditional antibiotic development [12,13]. Geographic disparities in MRSA prevalence—high in regions like the U.S. and China, lower in Scandinavia—reflect differences in healthcare practices and antimicrobial stewardship. The pathogen’s metabolic flexibility and intracellular persistence further complicate eradication, necessitating a shift from bactericidal approaches to innovative strategies [8,14,15].

**Figure 1:**
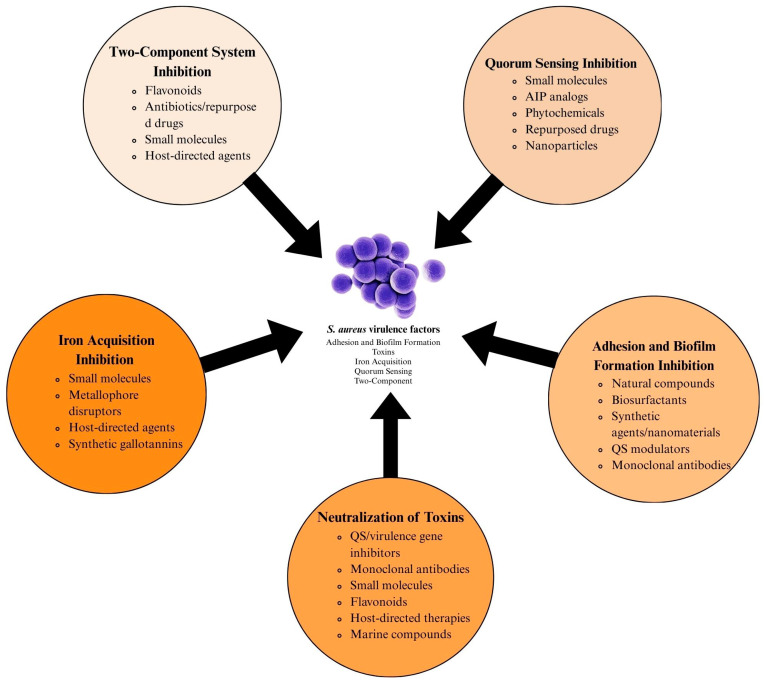
Multifaceted approach to *S. aureus* virulence factor inhibition.

To counter resistance and virulence, anti-virulence therapies aim to disarm *S. aureus* without exerting lethal pressure, thereby reducing resistance selection (Figure 1). QS inhibitors targeting the Agr system disrupt toxin production and biofilm dispersion, though the clinical translation is challenged by paradoxical biofilm reinforcement in chronic infections. Monoclonal antibodies (mAbs) neutralizing cytolytic toxins and immune-modulating therapies enhancing neutrophil recruitment offer complementary approaches yet face hurdles such as bacterial redundancy and host toxicity [16,17,18,19,20].

This review synthesizes recent advances in anti-virulence strategies against *S. aureus*, focusing on novel approaches inhibiting adhesion, biofilm formation, toxin production, quorum sensing, and iron acquisition. While we acknowledge that this article does not provide an exhaustive list of all molecules and strategies reported in the literature, we have endeavored to summarize the key findings from recent studies that highlight promising virulence inhibitors in *S. aureus*. By examining these strategies’ mechanisms, challenges, and therapeutic potential, we aim to underscore their role in mitigating antibiotic resistance and addressing the urgent need for alternatives to conventional antimicrobial therapies.

## 2. Inhibition of Adhesion and Biofilm Formation

Adhesion plays a critical role in the pathogenesis of *S. aureus*, facilitating its colonization on surfaces, evasion of host immune responses, and the establishment of biofilms [21]. Biofilm formation is significant in chronic and device-related infections, as it protects the bacteria from antimicrobial treatments and host immune defenses, making infections difficult to eradicate [22]. The ability of *S. aureus* to adhere to host tissues and medical devices significantly contributes to its virulence, with surface proteins such as fibronectin-binding proteins (FnBPs) and clumping factors (Clfs) mediating this adhesion to extracellular matrix components like fibrinogen and collagen [23]. This adhesion is especially critical in medical settings, where *S. aureus* can form biofilms on implants, leading to device-related infections such as surgical site infections [24,25]. Given the importance of adhesion in *S. aureus* infections, strategies to prevent this process are crucial for controlling biofilm-related infections, particularly those involving antibiotic-resistant strains like MRSA. Recent studies have highlighted various inhibitors, each targeting different stages of bacterial adhesion or biofilm formation mechanisms, providing promising avenues for therapeutic intervention [26,27,28].

Various natural compounds have been investigated for their ability to inhibit *S. aureus* biofilm formation (Table 1 and Table S1). Epigallocatechin gallate (EGCG), a polyphenolic compound, has shown significant anti-adhesive properties by functionalizing polymeric membranes and reducing bacterial adhesion by as much as 71% within four hours. The dose-dependent effect highlights the potential for optimizing EGCG concentration for therapeutic purposes [29]. Similarly, 10-hydroxy-2-decenoic acid (10-HDA), a fatty acid, disrupts the biofilm architecture by inhibiting extracellular polysaccharide production and significantly reducing biofilm biomass [30]. Another class of natural compounds, flavonoids such as rhodionin and isosakuranetin, inhibit biofilm formation by targeting sortase A (SrtA), an enzyme critical for bacterial attachment to host tissues [31,32].

The inhibition of SrtA is a common theme among several natural inhibitors. Verbascoside, hibifolin, and plantamajoside, all phenolic compounds derived from plants, disrupt the enzymatic activity of SrtA, reducing bacterial adhesion and biofilm formation [31,33,34,35,36]. These compounds present a promising alternative to traditional antibiotics, as they target bacterial adhesion mechanisms without promoting the development of resistance.

Other natural compounds, such as surfactin, a biosurfactant produced by *Bacillus subtilis*, also play a crucial role in biofilm inhibition. Surfactin reduces bacterial adhesion by modulating QS and disrupting extracellular polysaccharide production, vital for biofilm integrity [37,38,39]. Similarly, gallic acid (GA) has shown significant antibiofilm properties by inhibiting bacterial adhesion and aggregation and reducing extracellular polymeric substance (EPS) production at concentrations as low as 8 μg/mL [40].

In addition to natural compounds, synthetic agents and nanomaterials have been extensively studied for their anti-biofilm properties. Silver nanoparticles (AgNPs), particularly those synthesized using plant extracts, have shown remarkable efficacy in preventing bacterial adhesion and disrupting established biofilms [41,42]. For example, AgNPs from *Hellenia speciosa* rhizome extract eliminated up to 92.41% of *S. aureus* biofilms, effectively combating biofilm-related infections [43]. Similarly, chitosan-based nanoparticles, especially when combined with other natural compounds, have demonstrated the ability to inhibit biofilm formation in *S. aureus*, offering potential clinical and agricultural applications [44,45,46]. Gold nanoparticles functionalized with trehalose have also been explored for their anti-adhesive effects, showing reduced bacterial attachment to human endothelial cells [47].

Other synthetic agents, such as hexestrol (HXS), a nonsteroidal estrogen, inhibit biofilm formation by downregulating the expression of key biofilm-related genes and reducing extracellular polysaccharide production. HXS has demonstrated effectiveness against methicillin-sensitive and MRSA, making it a valuable candidate for combination therapies [48]. Furthermore, antimicrobial peptides such as DLL37-1 and LL37-1 have been shown to reduce bacterial adhesion and biofilm formation by disrupting the microbial surface components involved in the adhesion to host tissues [49].

The modulation of QS and gene expression represents another promising strategy for inhibiting biofilm formation in *S. aureus*. D-serine has been found to inhibit biofilm formation in MRSA by downregulating key biofilm-related genes and reducing bacterial adhesion to surfaces [50]. Similarly, when combined with antibiotics, acidic amino acids such as aspartic acid and glutamic acid disrupt the extracellular DNA meshwork within biofilms, enhancing the effectiveness of antibiotics and reducing biofilm formation [51]. Other compounds, such as S-342-3 and YH7, inhibit biofilm formation by downregulating the expression of genes involved in biofilm stability and adhesion, thus disrupting biofilm integrity [52,53].

Biosurfactants produced by microorganisms like *Lactobacillus helveticus* also offer an effective strategy for biofilm inhibition. These compounds reduce bacterial adhesion to surfaces and host cells and disrupt biofilm integrity by interfering with QS and gene expression, providing an eco-friendly alternative to chemical antibiofilm agents [54,55]. Additionally, bacteriocins and antimicrobial peptides such as P34 have reduced biofilm formation by interfering with bacterial cell wall synthesis and metabolic activity [56].

Nanomaterials have shown considerable promise in preventing bacterial adhesion and biofilm formation on medical devices [57]. Titanium alloys modified with vancomycin-bearing polymer brushes have significantly reduced bacterial adhesion and colonization in both in vitro and in vivo models [58,59]. Similarly, nano-apatites doped with noble metal ions exhibit enhanced anti-adhesive and anti-biofilm properties with low cytotoxicity, making them suitable for biomedical applications [60]. Chlorhexidine (CHX)-loaded nano-hydroxyapatite (nanoHA) biomaterials also reduce bacterial adhesion and biofilm formation on medical devices, providing controlled release profiles that ensure prolonged antibacterial effects while maintaining fibroblast viability, making them ideal for use in implantable medical devices [61].

Some inhibitors work by modulating the host response to bacterial infection, providing an indirect approach to combating biofilm-associated infections. Ensifentrine, which targets phosphodiesterase enzymes, has been shown to enhance lung endothelial and epithelial barrier function, thus protecting against MRSA-induced pneumonia and reducing inflammation [62]. Plant extracts such as *Vernonia condensata* have shown antimicrobial and anti-adhesive properties, mainly when combined with antibiotics like ampicillin. These suggest potential applications for preventing biofilm formation on food-contacting surfaces and medical devices [63].

Monoclonal antibodies targeting specific adhesins, such as the L-lectin module of *S. aureus* serine-rich repeat protein (SraP), have significantly reduced bacterial adhesion and invasion in human epithelial cells [64]. This innovative approach could block bacterial adhesion at the host–pathogen interface, offering a targeted strategy for preventing biofilm-associated infections [65,66].

Dual inhibition strategies, which target biofilm formation and bacterial virulence factors, have proven effective in reducing the pathogenicity of *S. aureus*. Compounds like quebrachitol (QBC) inhibit biofilm formation and suppress the production of virulence factors such as lipase and hemolysin, thereby reducing both bacterial adhesion and virulence [67]. This dual action makes QBC a promising candidate for therapeutic interventions aimed at both preventing biofilm formation and attenuating the pathogenicity of *S. aureus*.

**Table 1:** Examples of inhibitors and their mechanisms of action targeting adhesion in *S. aureus.*

Mechanism of Action	Inhibitor	Reference
Sortase A Inhibition		
Binds to and inhibits SrtA activity	Rhodionin	[32]
Reversible SrtA inhibition impairs SpA anchoring	Plantamajoside	[33]
Inhibits SrtA, reduces adhesion/biofilm	Hibifolin	[34]
Dual inhibition of SrtA and α-hemolysin	Isosakuranetin	[31]
Binds SrtA, induces conformational changes	2R,3R-dihydromyricetin	[35]
Inhibits SrtA and Hla, reduces adhesion	Daphnetin	[68]
Inhibits SrtB, reduces adhesion to lung cells	Coptisine	[69]
MSCRAMM Interaction Blockade		
Blocks ClfA/ClfB ligand-binding trenches	Allantodapsone	[70]
Inhibits MSCRAMM anchoring (SraP adhesin)	Anti-SraP L-lectin mAb	[64]
Biofilm Gene Downregulation		
Downregulates *icaA*/*icaD*	C1 and C2 (LMW compounds)	[71]
Downregulates *sarA*, *agr*, and *ica*	Quebrachitol	[67]
Downregulates *sarX* and PIA production	YH7	[53]
Suppresses *sarA*, *icaA*, and *icaD*	Gallic acid (GA)	[40]
Extracellular Matrix (ECM) Disruption		
Destabilizes ECM components	Fmoc-F	[72]
Disrupts eDNA meshwork	D-Asp/D-Glu	[51]
Reduces eDNA and metabolic activity	PASP and EO@PASP/HACCNPs	[73]
Surface Property Modification		
Alters surface hydrophilicity	Microalgal EPSs (Ta fraction)	[74]
Reduces adhesion via glycopolymer interactions	Trehalose-AuNPs	[75]
Membrane Integrity Disruption		
Disrupts membrane permeability	Vc-EAF (*Vernonia condensata*)	[63]
Nanoparticle-Mediated Disruption		
Downregulates PIA synthesis	Ag@Glu/Tsc NPs	[76]
Adhesion Gene Suppression		
Inhibits *clfA*, *sdrC*	DLL37-1 and LL37-1	[49]
Anti-Inflammatory and Anti-Adhesive		
Attenuates NF-κB pathway	Forsythiaside	[58]

Legend: Ag@Glu/Tsc NPs: silver nanoparticles functionalized with glucose/thiosemicarbazide. *agr*: accessory gene regulator. AuNPs: gold nanoparticles. ClfA/ClfB: clumping factors A and B. D-Asp/D-Glu: D-aspartic acid and D-glutamic acid. eDNA: extracellular DNA. ECM: extracellular matrix. EPSs: exopolysaccharides. GA: gallic acid. Hla: alpha-hemolysin. *icaA*/*icaD*: Intercellular adhesion operon genes. LMW: Low Molecular Weight. mAb: Monoclonal Antibody. MSCRAMM: Microbial Surface Components Recognizing Adhesive Matrix Molecules. NF-κB: Nuclear Factor kappa-light-chain-enhancer. NPs: Nanoparticles. PIA: Polysaccharide Intercellular Adhesin. *sarA*: staphylococcal accessory regulator A. *sarX*: a transcriptional regulator. SpA: staphylococcal protein A. SraP: staphylococcal surface adhesin P. SrtA/B: sortase A or B.

## 3. Neutralization of Toxins

The escalating threat of antibiotic resistance in *S. aureus* underscores the urgent need for innovative therapeutic strategies that circumvent traditional bactericidal approaches (Table 2 and Table S1). Central to *S. aureus* pathogenicity is its arsenal of virulence factors, particularly toxins such as α-hemolysin, Panton–Valentine leukocidin (PVL), Phenol-Soluble Modulins (PSMs), and exfoliative toxins, which disrupt host cell membranes, lyse immune cells, and drive severe infections ranging from necrotizing pneumonia to toxic shock syndrome [1,77].

**Table 2:** Selected inhibitors and their mechanisms of action targeting *S. aureus* toxins.

Category	Inhibitor	Mechanism of Action	Reference
1. Toxin Neutralization and Superantigen Interaction Blockade	Bispecific scFv MB102a	Allosterically inhibited SEB-TCR complex formation.	[78]
	Computational protein binder	Bound SEB’s MHC-II domain, inhibiting the inflammatory response.	[79]
	Hm0487 monoclonal antibody	Blocked SEB interaction with TCR/MHC-II; enhanced phagocytosis.	[80]
	Human cathelicidin LL-37	Bound LukS, inhibiting PVL toxin.	[81]
	LXY8 monoclonal antibody	Blocked SEB-TCR interaction	[82]
	Monoclonal antibody YG1	Neutralized Hla pore formation by targeting amino acids 205–212.	[83]
	pSEB116-132 peptide	Blocked SEB binding to CD28, reducing inflammation.	[84]
	scFv MS473	Neutralized TSST-1, preventing lethal shock.	[85]
2. Staphyloxanthin and Pigmentation Inhibition	AS8 (thymol-isatin hybrid)	Inhibited staphyloxanthin; reduced biofilm formation.	[86]
	Patuletin	Bound CrtM, inhibiting staphyloxanthin and biofilm formation.	[87]
	Terbinafine	Inhibited staphyloxanthin biosynthesis; disrupted biofilm.	[88]
3. Membrane Integrity Disruption and ROS Generation	Ga^3+^CHP (photoactivated)	Generated ROS via photodynamic inactivation, reducing toxin production.	[89]
	MSI-1 peptide	Disrupted membrane integrity; reduced STX synthesis.	[90]
	Rosmarinus officinalis/Myrtus communis oils	Induced oxidative stress; reduced catalase activity.	[91]
4. Coagulase and Fibrin Formation Inhibition	Baicalein	Inhibited coagulase activity of vWbp; enhanced penicillin G efficacy.	[92]
	Galangin	Bound vWbp inhibited fibrin formation and synergized with latamoxef.	[93]
	Sinensetin	Bound Coa residues (R73A, R204A), inhibiting coagulation.	[94]
5. Protease and Enzyme Inhibition	Ayanin	Inhibited ClpP; reduced virulence factors.	[95]
	Phenyl esters (MAS-19, MAS-30)	Inhibited ClpXP protease; reduced toxin production.	[96]
	Tamarixetin	Inhibited ClpP hydrolytic activity; suppressed virulence factors.	[97]
6. Immune Response Modulation	Anandamide (AEA)	Modulated miRNAs to increase anti-inflammatory molecules (ARG1, TGF-β2).	[98]
	Δ9-Tetrahydrocannabinol	Suppressed cytokines via CB2 receptor-induced Tregs and MDSCs.	[99]
	IBT-V02 vaccine	Generated neutralizing antibodies against multiple toxins.	[100]

Legend: ARG1: Arginase 1; *agrA*: accessory gene regulator A; CB2: Cannabinoid receptor type 2; ClpP: Caseinolytic protease P; ClpXP: Caseinolytic protease XP complex; Coa: Coagulase; Ga^3+^CHP: Gallium(III)-chlorin e6 photoconjugate; Hla: alpha-hemolysin; IBT-V02: vaccine targeting multiple S. *aureus* toxins; LL-37: human cathelicidin; MDSCs: myeloid-derived suppressor cells; MHC-II: major histocompatibility complex class II; miRNAs: microRNAs; PVL: Panton–Valentine leukocidin; ROS: reactive oxygen species; scFv: single-chain variable fragment; SEB: staphylococcal enterotoxin B; STX: staphylococcal toxins; TCR: T-cell receptor; TGF-β2: transforming growth factor beta 2; TSST-1: toxic shock syndrome toxin-1; vWbp: von Willebrand factor-binding protein.

### 3.1. Inhibition of Hemolysin Production

*S. aureus* is a formidable pathogen whose virulence is significantly attributed to the production of hemolysins, particularly alpha-hemolysin (Hla), which disrupts host cell membranes and exacerbate infection [101]. Recent research highlights diverse approaches that inhibit hemolysin production or activity, broadly categorized into mechanisms involving QS and virulence gene regulation, direct toxin neutralization, synergistic combinations with antibiotics, and host-directed interventions.

A prominent strategy involves disrupting QS and virulence gene regulatory pathways. Compounds such as 10-HDA inhibit biofilm formation and hemolysis by downregulating critical regulators like *sarA*, *agrA*, and *hla* genes [30]. Similarly, FDA-approved drugs (candesartan, domperidone, and miconazole) reduce hemolysin production by suppressing *hla* and *icaA* expression [102]. Ibuprofen and simvastatin diminish virulence factor expression, including Hla, by interfering with the *agr* system [32,103]. The pleuromutilin derivative 14-O-[(4,6-Diamino-pyrimidine-2-yl) thioacetyl] mutilin (DPTM) and the flavonoid 7,8-DHF attenuate Hla synthesis by targeting *agrA* and RNAIII, key components of the QS cascade [104,105]. Petroselinic acid and artesunate similarly suppress *agrA* and *hla*, disrupting toxin production [106,107]. Zinc sulfate and citral impair biofilm-associated genes (*icaA*, *icaD*) while concurrently reducing hemolytic activity, linking biofilm disruption to virulence attenuation [108,109]. Transcriptional inhibitors like nusbiarylins and ClpXP-targeting phenyl esters (MAS-19, MAS-30) further underscore the efficacy of blocking virulence gene expression [96,110].

Directly targeting the hemolysin structure and function represents another critical approach. Monoclonal antibodies, such as YG1, neutralize Hla by binding its pore-forming domain, preventing host cell lysis [83]. Small molecules like piceatannol and myricetin bind Hla, inhibiting oligomerization and pore formation [111,112]. Lysine disrupts Hla oligomerization, while iturins from *Bacillus velezensis* physically block hemolysin activity [19,113]. Theaflavin TF3 reduces Hla secretion and stabilizes host cell junctions, offering dual protection [114]. Quercetin modifies erythrocyte membranes to resist toxin insertion, illustrating a host-directed mechanism [115].

Synergistic strategies combining anti-hemolysins with antibiotics enhance therapeutic outcomes. Ibuprofen and 7,8-DHF potentiate vancomycin’s efficacy, improving wound healing and survival in infection models [103,104]. Quercetin augments ciprofloxacin and gentamicin by disrupting biofilms and virulence gene expression [116]. Zinc sulfate and iclaprim depolymerize biofilms, restoring antibiotic susceptibility [108,117]. These combinations mitigate resistance evolution by reducing selective pressure on bacterial growth.

Host-directed therapies focus on mitigating toxin-induced damage. DPTM inhibits NF-κB and inflammatory cytokines, alleviating MRSA-induced cell injury [105]. TF3 preserves keratinocyte integrity and reduces pro-inflammatory mediators, while myricetin suppresses MAPK/NF-κB pathways to limit lung injury [112,114]. Azithromycin diminishes hemolytic activity without bactericidal effects, potentially reducing inflammatory sequelae [118,119]. Monoclonal antibody cocktails neutralizing Hla and leukocidins significantly improve survival in septic shock models [120].

### 3.2. Inhibition of Enterotoxins

Staphylococcal enterotoxins (SEs) are pivotal virulence factors produced by *S. aureus*, contributing to severe pathologies such as food poisoning, toxic shock syndrome, and immune-mediated tissue damage [121,122]. These superantigens evade conventional immune responses by binding major histocompatibility complex class II (MHC-II) molecules and T-cell receptors (TCR), triggering disproportionate cytokine release and epithelial barrier disruption [123]. Recent advances in therapeutic strategies targeting enterotoxins have focused on direct neutralization, suppression of toxin production, immune modulation, and using natural or synthetic compounds, complemented by innovations in diagnostic detection.

Monoclonal antibodies (mAbs) and engineered proteins represent a cornerstone in neutralizing enterotoxins. The human mAb Hm0487 binds a linear epitope (SEB138–147) on staphylococcal enterotoxin B (SEB), inhibiting its interaction with TCR and MHC-II, thereby neutralizing superantigenic activity and cytokine storms [80]. Similarly, mAb LXY8 targets SEB residues 176EFNN179, blocking TCR binding with a high affinity (KD = 0.525 nM) and protecting against lethal shock in murine models [82]. Bispecific antibodies, such as the scFV MB102a, combine paratopes from distinct antibodies to allosterically disrupt SEB-TCR interactions, demonstrating enhanced neutralization efficacy [78]. Nanobodies, including Nb8, leverage unique complementarity-determining regions to block SEB-MHC-II binding, reducing inflammatory responses [79]. The SEB mimetic peptide pSEB116–132 also competitively inhibits SEB binding to CD28, restoring intestinal epithelial integrity by mitigating cytokine-driven NF-κB and STAT3 activation [84]. These strategies highlight the potential of biologics to sequester toxins and prevent immune activation directly.

Targeting regulatory pathways governing enterotoxin synthesis offers an alternative therapeutic avenue. Phenazopyridine hydrochloride (PP-HCl) inhibits the SaeRS two-component system, competitively binding SaeS kinase to reduce the phosphorylation-dependent expression of toxic shock syndrome toxin-1 (TSST-1) and SEB [124]. Photodynamic inactivation using gallium porphyrin (Ga^3+^CHP) suppresses *sec* and *tst* gene expression via reactive oxygen species, diminishing enterotoxin C (SEC) and TSST-1 production while preserving host cell viability [89]. Natural compounds, such as EGCG, downregulate *sea*, *icaA*, and QS genes, reducing staphylococcal enterotoxin A (SEA) secretion and biofilm formation [125]. Similarly, *Chrysanthemum zawadskii* essential oil inhibits *mecA* and *agrA*, attenuating MRSA enterotoxin production [126]. These approaches disrupt virulence gene networks, curbing toxin synthesis at the transcriptional level.

Modulating host immune responses to SEs can mitigate pathological inflammation. Blocking the 2B4 receptor on eosinophils prevents SEB-induced activation, reducing peritoneal inflammation and cytokine release in murine models [127]. Cannabinoids, including anandamide (AEA) and delta-9-tetrahydrocannabinol (THC), suppress SEB-driven cytokine storms by upregulating anti-inflammatory mediators (e.g., IL-10, TGF-β) and promoting regulatory T-cell expansion via miRNA modulation [98,99]. Vaccination strategies, such as the mRNA-based mSEB vaccine and multicomponent toxoid IBT-V02, elicit robust neutralizing antibodies against SEB, TSST-1, and other toxins, conferring protection against primary and recurrent infections [100,128]. These interventions rebalance immune responses, limiting tissue damage during infection.

Phytochemicals and prebiotics exhibit multifaceted anti-enterotoxic properties. Naringenin from avocado binds Staphylococcal enterotoxin-like X (SElX), neutralizing its superantigenicity and MRSA biofilm formation [129]. Non-digestible oligosaccharides (NDOs) and short-chain fatty acids (SCFAs) block enterotoxin adherence to host cells and promote gut microbiota resilience, indirectly suppressing pathogenicity [130]. Engineered chromones, optimized for binding TSST-1, demonstrate high solubility and permeability, offering synthetic alternatives to counteract toxin-mediated shock [131]. Such compounds exemplify the synergy between natural and synthetic approaches in toxin inhibition.

### 3.3. Inhibition of Staphyloxanthin Production

Staphyloxanthin (STX) production inhibition in *S. aureus* represents a promising avenue for developing anti-virulence strategies against this pathogen. Staphyloxanthin, a carotenoid pigment, is critical in protecting *S. aureus* from oxidative stress and host immune defenses, thus contributing significantly to its virulence [132]. Several compounds and strategies have been identified that specifically target the biosynthesis of STX by inhibiting key enzymes involved in its production, such as CrtM (Dehydrosqualene synthase) and CrtN (Dehydrosqualene desaturase) [133].

One common approach to inhibiting STX production involves the disruption of carotenoid biosynthesis enzymes. Patuletin, a natural flavonoid, has been shown to bind effectively to the CrtM enzyme, which is essential for the final step of STX synthesis. Molecular docking and molecular dynamics simulations have revealed a strong binding affinity between Patuletin and CrtM, significantly reducing STX production by 53% and 46% in different *S. aureus* isolates [87]. This reduction in STX diminishes the pathogen’s resistance to oxidative stress and enhances the susceptibility of *S. aureus* to human immune responses. Similarly, MSI-1, a peptide, has been shown to target both the membrane and carotenoid biosynthesis in *S. aureus*. MSI-1 disrupts bacterial membrane integrity by binding to lipoteichoic acid (LTA) and further interferes with the CrtN enzyme, which is involved in the early stages of carotenoid biosynthesis [90]. This dual mechanism significantly reduces STX levels, weakening the bacteria’s defense against oxidative stress and immune system attack. Moreover, MSI-1 exhibits synergistic effects when combined with vancomycin, suggesting that it could be developed as a therapeutic agent that reduces bacterial viability and targets virulence factors, such as STX.

Terbinafine, traditionally used as an antifungal agent, has also shown promising results in inhibiting STX production. By interfering with the CrtN enzyme, terbinafine significantly reduces STX synthesis dose-dependently [88]. This reduction in STX, along with its effects on biofilm formation and bacterial surface properties, makes terbinafine a potent anti-virulence agent. Celastrol, a compound derived from *Tripterygium wilfordii*, represents another promising inhibitor of STX biosynthesis. By targeting CrtM, celastrol causes an accumulation of farnesyl diphosphate, a precursor in the carotenoid pathway, thereby inhibiting STX production [134]. This inhibition reduces the golden pigment of *S. aureus* and increases bacterial susceptibility to oxidative stress and immune killing.

Rutin-loaded chitosan nanoparticles (Rut-CS NPs) have been developed as a novel delivery system for flavonoid rutin, which shows potent anti-virulence effects. These nanoparticles inhibit STX production in *S. aureus*, damaging the bacterial membrane and increasing DNA leakage [135]. Rut-CS NPs also enhance the bacterium’s susceptibility to hydrogen peroxide, weakening its defenses against oxidative stress.

Other compounds, such as thymol-isatin hybrid and chlorothymol, have also demonstrated efficacy in inhibiting STX production. Chlorothymol, a thymol derivative, reduces STX levels and biofilm formation at sub-minimal inhibitory concentrations [136]. Its synergy with oxacillin, particularly against MRSA, enhances its effectiveness as a therapeutic agent.

### 3.4. Inhibition of Coagulase Production

The inhibition of coagulase (Coa) in *S. aureus* represents a promising therapeutic strategy for addressing infections caused by this pathogen, which is notorious for its ability to evade immune responses and promote pathogenicity through the activation of prothrombin and fibrin formation. Coagulase, a key virulence factor in *S. aureus*, plays a pivotal role in blood clotting and biofilm formation, contributing significantly to the bacterial survival and persistence within the host [137,138]. Recent studies have explored various natural compounds as inhibitors of Coa activity, shedding light on their mechanisms and therapeutic potential.

Several flavonoids have been identified as potent inhibitors of Coa, highlighting their ability to selectively target this virulence factor without significantly affecting bacterial growth. For instance, isovitexin, a natural flavonoid, could bind to Coa directly, inhibiting its activity and preventing fibrin formation. Key amino acid residues such as Y188, V191, N267, and P268 were identified as critical for the interaction between isovitexin and Coa, providing valuable insights for designing anti-virulence therapies [139]. Similarly, isoquercitrin, another flavonol, inhibited Coa activity by binding to residues Asp-181 and Tyr-188, which are crucial for Coa’s ability to activate prothrombin. In vitro and in vivo studies have demonstrated that isoquercitrin significantly reduces the bacterial burden, alleviates lung inflammation, and improves survival rates in mice infected with *S. aureus* [140]. Another flavonoid, baicalein, derived from the Chinese herb *Scutellaria baicalensis*, has also been found to inhibit the coagulase activity of the von Willebrand factor-binding protein (vWbp), a similar virulence factor in *S. aureus*. Baicalein’s inhibitory effect on vWbp coagulase activity led to significant improvements in survival rates in a mouse pneumonia model, underscoring its potential as an anti-virulence agent. Additionally, baicalein enhanced the efficacy of penicillin G, suggesting that it could serve as a complementary therapeutic agent in combination with traditional antibiotics [92]. The ability of baicalein to inhibit coagulase while enhancing the effects of antibiotics represents a dual approach to combating *S. aureus* infections, particularly those caused by multidrug-resistant strains.

Sinensetin, another natural compound, was also shown to inhibit Coa-induced coagulation and biofilm formation, further emphasizing the role of flavonoids in targeting this virulence factor. Molecular docking and thermal shift assays revealed that sinensetin binds directly to Coa, enhancing its thermal stability and preventing its function in blood coagulation. Point mutation experiments identified key binding sites on Coa, including residues R73A-Coa and R204A-Coa. In vivo studies demonstrated that sinensetin treatment reduced tissue damage, lowered inflammation, and improved survival in both *Galleria mellonella* and mouse models of pneumonia when combined with oxacillin [94]. These results highlight sinensetin’s potential as a therapeutic agent in treating *S. aureus* infections.

Furthermore, galangin, a flavonoid with similar properties, has been reported to inhibit the coagulase activity of vWbp in *S. aureus*. Galangin’s binding to vWbp significantly increases its thermal stability and reduces its ability to promote fibrin formation, a critical process for *S. aureus* virulence. In vivo studies demonstrated that galangin improved survival rates and reduced bacterial loads and inflammation in a mouse model of *S. aureus*-induced pneumonia. Additionally, the combination of galangin with latamoxef, an antibiotic, enhanced therapeutic effects, highlighting the potential of galangin in combination therapies against *S. aureus* infections [93].

### 3.5. Inhibition of Other Toxins

Several studies have explored various compounds and natural products targeting different toxin production and expression mechanisms, presenting promising avenues for therapeutic intervention.

Ayanin, a natural flavonoid isolated from *Callicarpa nudiflora*, has been identified as a potent inhibitor of Caseinolytic protease (ClpP), a crucial virulence factor in *S. aureus*. ClpP plays a significant role in stress survival, antibiotic resistance, and pathogenesis, and its inhibition by ayanin was shown to reduce the expression of multiple virulence factors, including hemolysin (hla), PVL, and others involved in biofilm formation and immune evasion [95]. Similarly, tamarixetin, another flavonoid, also targets ClpP, inhibiting its hydrolytic activity and suppressing the transcription of virulence factors such as *hla, agr*, and *pvl*. Both compounds highlight the potential of ClpP inhibition in reducing toxin production without directly affecting bacterial growth, suggesting a mechanism that could reduce pathogenicity without contributing to resistance [97].

Further strategies targeting the inhibition of toxin production include using essential oils from *Rosmarinus officinalis* (rosemary) and *Myrtus communis* (myrtle). These oils demonstrated significant anti-virulence activity, particularly in reducing the production of hemolysin, DNase, and protease, which are critical to *S. aureus*’s ability to evade host defenses. The combined oils inhibited biofilm formation and triggered oxidative stress in bacterial cells, leading to reduced catalase activity, further impeding the bacterium’s ability to neutralize reactive oxygen species (ROS) [91]. This approach underscores the potential of using natural products as adjunctive therapies to mitigate toxin-related damage during *S. aureus* infections.

Marine-derived compounds, such as naphto-γ-pyrones (NGPs) from *Aspergillus welwitschiae*, have also been shown to inhibit key virulence factors of *S. aureus*, including biofilm formation and hemolysis, by directly targeting toxins like hemolysin. NGPs reduced hemolytic activity by approximately 80% and, when combined with vancomycin, enhanced the antibiotic’s efficacy in treating *S. aureus* infections in vivo. The ability of NGPs to interfere with toxin production without inhibiting bacterial growth positions them as valuable candidates in the fight against antibiotic resistance [141].

Another notable approach focuses on inhibiting the PVL, a significant toxin responsible for the virulence of MRSA. Human cathelicidin LL-37 was found to bind strongly to the PVL subunits LukS and LukF, thus inhibiting the toxin’s cytotoxic effects and preventing pore formation in host neutrophils. In silico studies confirmed the potential of LL-37 to outcompete commercial PVL inhibitors, providing a promising therapeutic option for tackling MRSA infections, particularly in nosocomial settings [81]. Additionally, specific receptor antagonists targeting the C5aR receptor, such as avacopan, PMX205, and W-54,011, were found to mitigate the effects of PVL by reducing its cytotoxicity and inflammasome activation in monocytes. These findings suggest a broader strategy for targeting toxin activity via immune modulation [142]. Reyes-Robles et al. identified a novel approach for neutralizing the virulence of *S. aureus* by targeting its bicomponent leukocidins, causing immune cell lysis [143]. A glycine-rich motif in these toxins is crucial for pore formation in host cell membranes, leading to cell lysis. Deleting this motif renders the toxins unable to form pores, exerting a dominant-negative effect that blocks the activity of both native and heterologous toxin subunits [144]. This strategy effectively neutralizes the cytotoxicity of various *S. aureus* toxins, including PVL, in vitro and in vivo, protecting against lethal challenges in animal models [145].

Triterpenoid acids isolated from *Schinus terebinthifolia* fruits demonstrated a novel mechanism of action by inhibiting QS in *S. aureus*. These compounds suppressed the expression of various virulence factors, including leucocidin A and δ-toxin, responsible for cytotoxicity and tissue damage. The inhibition of the accessory gene regulator (*agr*) system, a key regulator of virulence factor expression, further underscores the importance of disrupting bacterial communication pathways to reduce toxin production and pathogenesis in MRSA infections [146].

Rouha et al. investigated the neutralization of six *S. aureus* cytotoxins using two human monoclonal antibodies (mAbs), ASN-1 and ASN-2. ASN-1 targets alpha-hemolysin and four leukocidins (LukSF-PV, LukED, HlgAB, and HlgCB), while ASN-2 neutralizes the fifth leukocidin, LukGH. Their combination, ASN100, effectively neutralized the cytotoxic effects of these toxins in human neutrophils, monocytes, NK cells, and T-lymphocytes. Both antibodies were required to prevent cytotoxicity in *S. aureus*-infected human cells, preserving immune cell function and tissue integrity. Additionally, ASN100 provided complete protection in a 3D lung tissue model, preventing epithelial damage caused by *S. aureus*. These findings suggest that ASN100 could be a promising therapeutic approach for preventing *S. aureus*-induced cell lysis and tissue damage, particularly in vulnerable populations such as mechanically ventilated patients [147].

The study by Zhou et al. explores the potential of myricetin, a natural flavonoid, as an inhibitor of Type II NADH dehydrogenase (NDH-2) in *S. aureus*, a key enzyme involved in the bacterium’s respiratory chain and energy production. NDH-2 has been identified as a promising target for antibacterial therapy, mainly because its inhibition disrupts the pathogen’s growth and virulence without affecting humans who lack this enzyme. Myricetin showed potent inhibitory activity with an IC50 of 2 µM and was effective against bacterial growth, with its activity antagonized by menaquinone-4 (MK-4), a substrate of NDH-2. Additionally, myricetin reduced the expression of enterotoxin SeA and inhibited hemolytic activity [148].

In another study, Shatalin et al. investigated the role of bacterial hydrogen sulfide (H_2_S) production in *S. aureus* and its potential as a target for enhancing antibiotic efficacy. The researchers identified cystathionine-γ-lyase (bCSE) as the primary enzyme responsible for H2S generation in *S. aureus*. The inhibition of bCSE by small molecules potentiated the effect of various bactericidal antibiotics, both In vitro and in vivo. This inhibition disrupted biofilm formation and reduced bacterial persistence, key factors in chronic infections. The study highlights the critical role of H_2_S in antibiotic tolerance and persister cell formation, suggesting that targeting the H_2_S biogenesis pathway can improve the effectiveness of existing antibiotics against persistent bacterial infections [149].

## 4. Iron Acquisition Inhibitors

Iron acquisition is a critical process for *S. aureus* pathogenesis, as iron is an essential cofactor for numerous metabolic and virulence pathways. Staphylococci have evolved multifaceted systems to scavenge iron from host sources (Table 3 and Table S1), including heme-sequestering proteins, ferrous iron transporters, metallophores, and siderophores [150,151]. Targeting these systems presents a promising therapeutic strategy to disrupt bacterial survival and virulence. Recent studies have explored diverse approaches to inhibit iron uptake in staphylococci, ranging from direct interference with iron transport machinery to the indirect modulation of the host iron metabolism or bacterial compensatory mechanisms.

**Table 3:** Examples of inhibitors targeting iron acquisition in *S. aureus.*

Inhibitor	Action Mechanism	Reference
C35*	Inhibits IsdB-Hb complex formation, preventing heme acquisition by *S. aureus*	[152]
Synthetic gallotannins (PGG, G4Glc, G4Man)	Inhibits *S. aureus* biofilm formation, interacts with QS systems, and exhibits antioxidant properties	[153]
Compound 9	Inhibits CntL, reducing intracellular iron, nickel, and cobalt concentrations under metal-limited conditions	[154]
HSGN-220, HSGN-218, HSGN-144	Inhibits DNA replication, modulates menaquinone biosynthesis, starves bacteria of iron by upregulating heme/siderophore proteins, and disrupts membrane potential without altering permeability	[155]
GW3965·HCl, PHT-427	Reduces FeoB enzymatic activity, bacterial growth, and virulence factors (e.g., staphyloxanthin); alters bacterial metabolism to reduce iron availability	[156]
Saikosaponin A (SSA)	It reduces inflammation markers (MPO, TNF-α, IL-1β) and inhibits *S. aureus*-induced ferroptosis by decreasing iron accumulation and enhancing GPX4 activity	[157]

Legend: CntL: a component of the S. aureus Cnt metal transporter system; FeoB: ferrous iron transporter B; G4Glc: galloylglucose (glucose-linked gallotannin derivative); G4Man: galloylmannose (mannose-linked gallotannin derivative); GPX4: glutathione peroxidase 4; Hb: hemoglobin; IL-1β: interleukin-1 beta; IsdB: iron-regulated surface determinant B; MPO: myeloperoxidase; PGG: pentagalloylglucose; QS: quorum sensing; TNF-α: Tumor Necrosis Factor alpha.

One prominent approach involves disrupting heme acquisition, a primary iron source for *S. aureus*. Cozzi et al. developed small-molecule inhibitors targeting the interaction between human hemoglobin (Hb) and the IsdB hemophore, essential for extracting heme from host hemoglobin. Through structure-based virtual screening and biochemical validation, compound C35* was identified as a potent inhibitor of the IsdB-Hb complex, exhibiting a dissociation constant of 0.57 ± 0.06 µM. By blocking this interaction, C35* prevents bacterial heme uptake without perturbing host iron homeostasis, offering a pathogen-specific antimicrobial strategy [152].

In contrast to heme acquisition, the ferrous iron transport system, mediated by the FeoB protein, represents another critical target. Shin et al. demonstrated the efficacy of FeoB inhibitors. GW3965·HCl and PHT-427 were shown to suppress FeoB enzymatic activity, reducing intracellular iron availability and impairing bacterial growth. Both compounds diminished virulence factors such as staphyloxanthin production and biofilm formation, enhancing bacterial susceptibility to oxidative stress and antibiotics. PHT-427 exhibited synergistic effects with conventional antibiotics and demonstrated safety in animal models, underscoring its potential for treating bovine mastitis. These findings collectively validate FeoB as a viable target for anti-staphylococcal therapies [156].

The metallophore-mediated metal acquisition system, particularly the cobalt and nickel transporter (Cnt) system, has also been explored. Luo et al. designed dual-site inhibitors targeting CntL, an enzyme critical for staphylopine biosynthesis. Compound 9, the most potent inhibitor, exhibited a Kd of 0.021 ± 0.004 µM and reduced intracellular iron, cobalt, and nickel levels under metal-limiting conditions [154]. This study provides the first chemical evidence that disrupting metallophore biosynthesis effectively starves *S. aureus* of essential metals, presenting a novel avenue for antibacterial development.

Multi-target agents represent another innovative strategy. Naclerio et al. investigated halogenated oxadiazole derivatives, such as HSGN-220, which induce iron starvation by upregulating bacterial heme and siderophore biosynthesis proteins—a compensatory response indicative of iron limitation. These compounds concurrently inhibit DNA replication and menaquinone biosynthesis, leading to metabolic disruption and membrane depolarization [155]. Their pleiotropic effects and low resistance development highlight their potential as robust antimicrobial agents against MRSA.

Host-directed therapies that modulate iron availability offer an indirect yet complementary approach. Zhao et al. demonstrated that Saikosaponin A (SSA) alleviates *S. aureus*-induced mastitis by inhibiting host ferroptosis, an iron-dependent cell death pathway. SSA reduced mammary iron accumulation, suppressed lipid peroxidation, and activated the SIRT1/Nrf2 antioxidant axis, limiting iron availability for bacterial utilization [157]. This mechanism mitigates host tissue damage and indirectly impairs bacterial iron acquisition.

Finally, interference with bacterial communal behaviors, such as biofilm formation, may indirectly disrupt iron scavenging. Hricovíniová et al. reported that synthetic gallotannins inhibit *S. aureus* biofilm formation at sub-inhibitory concentrations and interfere with QS. As biofilms facilitate iron sequestration and siderophore production in bacterial communities, their disruption could compromise iron acquisition efficiency, though further studies are needed to elucidate direct links to iron metabolism [153].

## 5. Anti-QS Strategies

Quorum sensing in *S. aureus*, primarily mediated by the accessory gene regulator (Agr) system, coordinates virulence and biofilm formation, presenting a strategic target for anti-virulence therapies (Table 4 and Table S1) [1,158]. The Agr system, comprising AgrC (a histidine kinase) and AgrA (a response regulator), activates virulence gene transcription upon autoinducing peptide (AIP) detection [159]. Recent advances in QS inhibition have elucidated diverse mechanisms, ranging from direct Agr component targeting to interference with global regulators and biofilm-associated pathways, leveraging natural, synthetic, and repurposed compounds to attenuate pathogenicity while minimizing resistance risks.

**Table 4:** Examples of inhibitors and their mechanisms of action targeting QS in *S. aureus*.

Inhibitor	Action Mechanism	Reference
Visomitin	Inhibits Agr system; reduces virulence factors (hemolysin, staphyloxanthin) and biofilm formation	[160]
*Bacillus subtilis*-derived peptides	Modulates Agr signaling inhibits biofilm formation and enhances antibiotic susceptibility	[161]
Ambuic acid	Suppresses AIP-I and δ-toxin production; inhibits QS-regulated virulence	[162]
*3Staphylococcus simulans* AIP-I	Blocks MRSA *agr* signaling; reduces toxin production and skin necrosis	[163]
*Staphylococcus warneri* AIPs	Inhibits MRSA *agr*-IV signaling; reduces toxin production	[164]
Carnosic acid, carnosol	Inhibits *agr*-mediated QS signaling; reduces RNAIII expression and virulence factors (PSMs, α-hemolysin)	[165]
Pyrazolopyrimidine, thiazolopyridine	Inhibits *agr*-mediated QS; suppresses Phenol-Soluble Modulin (PSM) production	[166]
Pyocyanin	Binds AgrA; downregulates *agrA*; and reduces biofilm formation and toxin production	[167]
Azan-7	Binds AgrA blocks interaction with P3 promoter and reduces hemolysis and biofilm formation	[168]
orrectCNP0238696	Binds AgrA (in silico); inhibits QS-regulated virulence	[169]
Flavuside B (FlaB)	Suppresses *agrA* expression; reduces virulence and inflammation	[170]
G5-QQ3 dendrimer	Binds AgrA; inhibits hemolysis and biofilm formation	[171]
Staquorsin	Binds AgrA; inhibits RNAIII transcription; reduces virulence factors and biofilm formation	[172]
Physalins (H, B, isophysalin B)	Binds AgrA DNA-binding site; suppresses virulence gene expression	[173]
Actinomycetales-derived metabolites	Inhibits AgrA; reduces hemolysis and virulence factor expression	[174]
Epoxide compounds (ambuic acid)	Inhibits AgrA; reduces hemolysis and biofilm formation	[175]
Bumetanide	Binds AgrA (Tyr-229); downregulates *hla*, *psmα*, and *lukS-PV*; and promotes wound healing	[176]
Eugenol	Inhibits AgrA phosphorylation; suppresses *agrA*, *agrC*, RNAIII, *hla*, and *seb*; and disrupts energy metabolism	[177]
MA01 rhamnolipid	Downregulates *agrA* and *agrC*; inhibits biofilm formation	[178]
Probiotic-derived metabolites	Downregulates *agrA*; reduces staphyloxanthin and α-hemolysin production	[179]
Carboxypyranoanthocyanins	Downregulates QS genes (*agrA*, RNAIII); inhibits biofilm formation	[180]
Shikonin	Downregulates QS genes (*agrA*, *RNAIII*); reduces biofilm formation	[181]
5-acetyl-4-methyl-2-(3-pyridyl) thiazole (AMPT)	Binds AgrA and SarA; inhibits biofilm formation and virulence factors (hemolysin, protease)	[182]
Gliptins (sitagliptin)	Downregulates QS genes (*agrA*, *sarA*); inhibits biofilm formation and toxin production	[183]
Juglone derivatives (e.g., resveratrol)	Binds AgrC (in silico); suppresses agr-regulated virulence	[184]
Macrocyclic QQ peptides (QQ-1 to QQ-4)	Competitively inhibits AgrC activation; blocks virulence gene expression	[185]
Peptidomimetics (PhPr(3Br)-Bnc3)	Inhibits *agr* signaling; reduces toxin production without affecting growth	[157]
Morin	Binds SarA, inhibiting DNA binding; reduces biofilm formation	[186]
Vaccenic acid	Downregulates QS genes (SarA); inhibits biofilm formation and virulence factors.	[187]

Legend: AMPT: synthetic thiazole derivative targeting AgrA/SarA; Agr: accessory gene regulator; AgrC: histidine kinase component of the Agr two-component system; AIP: autoinducing peptide; FlaB: flavuside B; *hla*: α-hemolysin gene; lukS-PV: subunit of Panton–Valentine leukocidin; MRSA: methicillin-resistant *S. aureus*; PSM: Phenol-Soluble Modulin; PhPr(3Br)-Bnc3: peptidomimetic inhibitor of Agr; QQ: Quorum Quenching peptides; QS: quorum sensing; RNAIII: effector RNA of the Agr system; SarA: staphylococcal accessory regulator A; *seb*: staphylococcal enterotoxin B; Tyr-229: tyrosine residue at position 229 in AgrA.

Inhibition of AgrA, the central transcriptional activator, has been achieved through structurally diverse compounds. Small molecules like Visomitin disrupt Agr signaling, reducing hemolysin production and biofilm formation in MRSA at sub-inhibitory concentrations [160]. Similarly, pyocyanin from *Pseudomonas aeruginosa* binds AgrA, suppressing protease and toxin expression [167]. Azan-7, an aza-derivative, selectively blocks AgrA-P3 promoter interactions, impairing hemolysis without resistance induction [168]. Carboxypyranoanthocyanins and repurposed gliptins downregulate *agrA* and RNAIII, attenuating toxin production [180,183]. Staquorsin, physalins, and Actinomycetales-derived Phenalinolactones further inhibit AgrA-DNA binding or ATPase activity, suppressing virulence [172,173,174]. Eugenol, a phytochemical, impedes AgrA phosphorylation, reducing enterotoxin synthesis [177].

Competitive inhibitors disrupt AgrC- and AIP-mediated signaling. AIP analogs from *Staphylococcus warneri* and *S. simulans* block AgrC activation, reducing dermo-necrosis in vivo [163,164]. Macrocyclic peptides (QQ-1 to QQ-4) and peptidomimetics like PhPr(3Br)-Bnc3 exhibit nanomolar affinity for AgrC, inhibiting Agr-I to Agr-IV subtypes [185,188]. Juglone derivatives, such as resveratrol, bind AgrC in silico, while rhamnolipids from *P. aeruginosa* downregulate *agrC* and biofilm genes [178,184].

The global regulator SarA, which stabilizes biofilms and enhances virulence, is inhibited by morin and vaccenic acid, reducing EPS synthesis and staphyloxanthin production [186,187]. Gliptins exhibit pleiotropic effects, downregulating *sarA*, *sigB*, and *icaA* to destabilize biofilms [183]. The thiazole derivative AMPT targets AgrA and SarA concurrently, impairing MRSA virulence [182]. Coumarin–chalcone C9 disrupts cyclic-di-GMP signaling, sensitizing biofilms to antibiotics [189]. *Enterococcus faecium* and *B. subtilis* metabolites suppress *agrA* and *luxS*, enhancing oxidative stress susceptibility [161,179].

Plant-derived phytochemicals demonstrate broad anti-QS activity. Shikonin suppresses *agrA* and RNAIII, disrupting polymicrobial biofilms [181]. Saudi Sidr and Sumra honey inhibit violacein and pyocyanin production, reducing MRSA biofilms by >68% [190,191]. Propolis triterpenes, pomegranate extracts, and rosemary carnosic acid attenuate ag signaling and toxin synthesis [165,192,193]. Marine-derived siphonocholin and actinobacterial extracts impair biofilm formation via QS regulatory proteins like BfmR [194,195].

Synthetic small molecules, including pyrazolopyrimidines and epoxide-containing compounds, inhibit AIP biosynthesis and AgrA binding [166,175]. Repurposed drugs like bumetanide and 5-fluorouracil (5-FU) suppress virulence genes and enhance antibiotic efficacy [196,197].

Nanoparticles enhance QS inhibition through targeted delivery and synergistic mechanisms. Lignin-capped silver nanoparticles (AgLNPs) downregulate QS genes and destabilize biofilms via membrane disruption [198]. The study by Alenazi et al. investigated the anti-QS activity of a poly-amidoamine (PAMAM) dendrimer loaded with a quorum quencher (QQ3) peptide, which functions as a histidine kinase inhibitor against MRSA. The study found that the G5-QQ3 complex effectively inhibited hemolysis at concentrations of 10 µM for MRSA and 3 µM for *S. aureus*, suggesting a strong anti-toxin effect. The complex also demonstrated biofilm inhibition and eradication across different *agr* mutant strains, with inhibition rates ranging from 60% to 72%. [171]. The study by Hu et al. explores a dual-action strategy combining photothermal therapy and QS inhibition to enhance titanium (Ti)--based implants’ antibacterial properties and osseointegration. Luteolin-loaded mesoporous polydopamine nanoparticles and photothermal therapy achieve synergistic biofilm eradication [199].

## 6. Anti-Two-Component Strategies

Two-component regulatory systems (TCSs) are conserved bacterial signaling pathways that enable pathogens like *S. aureus* to adapt to environmental stresses and regulate virulence factor production, including toxins, immune evasion proteins, and biofilm formation [200]. The inhibition of TCSs in *S. aureus* has emerged as a promising strategy to attenuate virulence and combat antibiotic resistance (Table 5 and Table S1), particularly in MRSA [201]. Among the TCSs studied, the SaeRS and ArlRS systems have been extensively targeted due to their central roles in regulating virulence factors, including hemolysins, leukotoxins, and biofilm-associated proteins.

**Table 5:** Selected inhibitors targeting two-component systems in *S. aureus.*

Inhibitor	Action Mechanism	Reference
SKKUCS	Inhibits SaeS kinase activity by blocking ATP binding	[202]
Norlichexanthone	Interferes with AgrA-DNA binding and represses SaeRS-regulated genes	[203]
NH125	Noncompetitively inhibits VraS autophosphorylation	[204]
Dextran sodium sulfate (DSS)	Downregulates SaeRS-regulated virulence genes (toxins, adhesins)	[205]
I-modulia^®^	Enhances DSS-mediated suppression of virulence factors (secretion systems and exo-proteases)	
Five lead compounds (unnamed)	Bind to GraR dimerization interface, disrupting DNA interaction	[206]
Resveratrol	Downregulates *saeRS* expression, reducing virulence factor production	[207]
Phenazopyridine hydrochloride (PP-HCl)	Inhibits SaeS kinase activity, reducing SaeR phosphorylation	[124]
Fatty acids (FA)	Indirect inhibition via FA accumulation, suppressing SaeS activation	[208]
PMI-5	Targets ATP-binding domain of histidine kinases, suppressing virulence-associated TCS activation	[209]
HR3744, SAV13	Disrupts SaeR DNA-binding activity	[210]
TST1N-224	Disrupts VraRC-DNA complex formation, targeting DNA-binding residues	[211]
Gambogic acid (GA), Neogambogic acid (NGA)	Downregulates *saeRS*-regulated virulence genes (hemolysins, leukocidins, and fibrinogen-binding proteins)	[212]
Fenoprofen	Prevents SaeR-DNA binding, repressing virulence factors	[213]
3,4′-DMF	Directly inhibits ArlS autophosphorylation	[214]
Xanthoangelol B	Binds to SaeS, inhibiting histidine kinase activity	[215]
Verteporfin	Interferes with redox sensing via C227 residue in GraS	[216]
Sulfonamide derivative (compound 5)	Binds to ATP-binding domain of VraS	[217]
3,4′-Dimethoxyflavone derivatives (compound 17)	Inhibits ArlRS-dependent gene regulation; suppresses β-lactam resistance via GraRS or *mecA*-PBP2a	[218]
Antisense yycG RNA (ASyycG)	Downregulates YycFG pathway activity, reducing biofilm formation and virulence gene expression	[219]

Legend: AgrA: accessory gene regulator A; ArlRS: TCS involved in regulating bacterial adhesion and virulence; ArlS: sensor kinase of the ArlRS TCS; ASyycG: antisense RNA targeting yycG mRNA; C227: cysteine residue at position 227 in GraS; DMF: 3,4′-Dimethoxyflavone; GraR: response regulator of the GraSR TCS; GraS: sensor kinase of the GraSR TCS; *mecA*-PBP2a: methicillin resistance gene (*mecA*) and its protein product, penicillin-binding protein 2a; SaeRS: two-component system; SaeS: sensor kinase component of the *S. aureus* SaeRS; TCS: two-component system; VraRC: complex of VraR (response regulator) and DNA-binding components; VraS: sensor kinase of the VraSR TCS; YycFG: essential TCS for cell wall metabolism and biofilm formation in *S. aureus*.

The ArlRS TCS, governing QS and biofilm dynamics, is inhibited by flavonoids such as 3,4′-dimethoxyflavone (3,4′-DMF), which directly blocks ArlS autophosphorylation, reducing leukotoxin production in MRSA [214]. Structural analogs like compound 17, 3,4′-dimethoxyflavone derivatives enhance ArlRS inhibition and reverse β-lactam resistance [218]. Tilmicosin suppresses ArlS kinase activity (IC~50~ = 1.09 µM), synergizing with oxacillin to disrupt biofilms [220]. The study by Kwiecinski et al. (2022) explores the therapeutic potential of inhibiting the *ArlRS* two-component regulatory system in *S. aureus*, including MRSA. The research identifies two novel inhibitors, 3,4′-dimethoxyflavone (3,4′-DMF) and homopterocarpin (HPC), which block *ArlRS* signaling without affecting bacterial growth. In vitro, kinase assays confirm that 3,4′-DMF directly inhibits *ArlS* autophosphorylation, whereas HPC operates through an alternative mechanism [214]. Maleimide-based PMI-5 targets the ATP-binding domain of histidine kinases, including ArlS, reducing hemolysis and MRSA pathogenicity [209].

The SaeRS TCS, controlling toxins and immune evasion factors, is inhibited through diverse strategies. Phenazopyridine hydrochloride (PP-HCl) impairs SaeS autophosphorylation, reducing toxic shock syndrome toxin-1 (TSST-1) production [124]. Xanthoangelol B and its derivative PM-56 suppress α-hemolysin and aureolysin expression by blocking SaeS kinase activity [215]. Gambogic acid and neogambogic acid downregulate SaeRS-dependent hemolysins and leukocidins, impairing biofilm formation [212]. Fatty acid kinase indirectly modulates SaeRS via lipid accumulation, reducing toxin transcription [208]. Small molecules HR3744 and SAV13 disrupt SaeR-DNA binding, silencing *hla* and *lukPV* genes [210]. The study by Duan et al. investigated the effect of resveratrol on *S. aureus* virulence, explicitly targeting the SaeRS two-component system. The findings revealed that sub-inhibitory concentrations of resveratrol significantly downregulate *saeRS* expression, leading to a marked reduction in alpha-hemolysin production [207]. Norlichexanthone broadly represses SaeRS-dependent toxins (e.g., *hla* and *hlb*) and disrupts Agr QS [203]. Dextran sodium sulfate and SKKUCS inhibit SaeRS-mediated adhesins and exoproteases without bactericidal effects [202,205].

Verteporfin inhibits GraS in the GraXRS system by disrupting redox sensing via residue C227, indirectly suppressing SaeRS-regulated immune evasion proteins and enhancing neutrophil-mediated clearance [216]. The VraSR system, critical for β-lactam resistance, is targeted by sulfonamide derivatives (N1, N3, N9, and N10) and TST1N-224, which destabilize VraR-DNA interactions, reversing *pbpB* and *blaZ* expression [211,217]. NH125 blocks VraS autophosphorylation, restoring carbenicillin and vancomycin susceptibility [204]. The study by Lee et al. identifies small molecules that inhibit the VraTSR two-component system in MRSA, thereby potentiating the activity of oxacillin [221].

Fenoprofen repurposed as a SaeR inhibitor prevents DNA binding, repressing >20 virulence factors, including biofilms [213]. Similarly, HR3744 and SAV13 block SaeR’s transcriptional activity [210]. In the VraSR system, TST1N-224 binds VraR’s α9/α10-helices (K~D~ = 23.4 µM), inhibiting promoter interactions [211].

PMI-5 exhibits broad activity against virulence-associated TCS, including SaeRS and ArlRS [209]. Calvez et al. reported that transcriptomic analyses reveal that dextran sodium sulfate (DSS) alone downregulates key virulence-related genes controlled by the SaeRS two-component system, including those responsible for host immune evasion (scn and sbi), exotoxins (α-toxin and γ-toxin), and adhesins (map and emp). The combination of DSS and I-modulia^®^ further enhances the suppression of virulence factors, extending its inhibitory action to secretion systems and exo-proteases [205]. The study by Wu et al. explored the impact of antisense RNA (asRNA) regulation on the YycFG two-component regulatory system in *S. aureus*, particularly in MRSA strains associated with chronic osteomyelitis. By overexpressing antisense yycG RNA, the study successfully downregulated YycFG pathway activity, significantly reducing biofilm formation and decreasing virulence-related gene expression [222].

## 7. Strain-Specific Challenges in Anti-Virulence Therapeutics

The reviewed anti-virulence strategies demonstrate variable efficacy against *S. aureus* strains, reflecting the pathogen’s genetic and phenotypic diversity. For instance, propolis triterpenoids exhibit strain-specific activity, inhibiting biofilms in *S. aureus* but lacking efficacy against other *Staphylococcal* species [193]. Similarly, MA01 rhamnolipid downregulates *agrA*, *agrC*, and *icaA*/*D* in MRSA, yet its high effective concentrations (30–120 mg/mL) may limit its utility against heterogeneous clinical isolates [178]. Strain-dependent variability is further evident in therapies targeting QS: Siphonocholin (Syph-1) binds BfmR, a regulator conserved across *S. aureus* lineages, achieving 60% biofilm inhibition [194], whereas *Staphylococcus warneri* AIPs show differential potency (AIP-II > AIP-I) against MRSA *agr* types [164].

Control mechanisms often exploit conserved virulence pathways. For example, visomitin and resveratrol inhibit the Agr system, which is critical for toxin production in most *S. aureus* strains, albeit requiring sub-MIC or supraphysiological concentrations for bactericidal effects [160,184]. Broad-spectrum approaches, such as metal oxide nanoparticles (80–88% biofilm disruption) and lignin-capped AgNPs (downregulating *luxR*), bypass strain-specific QS variability by targeting structural biofilm components [198,223]. However, their lack of QS inhibition limits their precision and targeted efficacy.

Combinatorial strategies are emerging to address this variability. The MPDA-LUT@CaP implant coating integrates photothermal therapy (PTT) and QS inhibition, achieving 95.59% antibacterial rates across diverse MRSA strains [224]. Similarly, synthetic gallotannins disrupt biofilms at sub-MIC concentrations while suppressing QS, offering dual-action efficacy [153]. Clinical translation will require prioritizing multi-target agents—such as HSGN-220/218, which disrupt DNA replication, iron homeostasis, and membrane potential—to counteract strain-specific resistance mechanisms [155].

Anti-virulence candidates must be validated against a phylogenetically diverse panel of *S. aureus* strains, including MDR and small-colony variants (SCVs), to ensure robust development. Compounds exhibiting dual QS and biofilm inhibition, such as MA01 rhamnolipid and Syph-1, should be prioritized to mitigate strain-specific evasion. Furthermore, hybrid therapies combining anti-virulence agents—such as Agr inhibitors—with immunomodulators may enhance host–pathogen specificity and improve therapeutic outcomes.

Although preclinical studies highlight the potential of anti-virulence strategies, clinical validation remains limited. Bumetanide, a repurposed diuretic, reduced ulceration in vivo via AgrA inhibition (70% at 0.1 µM) without observable toxicity, indicating promise for topical formulations [196]. Similarly, fenoprofen—an FDA-approved NSAID—attenuated biofilm pathogenicity in implant models, though chronic use may carry the risk of NSAID-associated side effects [213]. Notably, the SaeR inhibitor SKKUCS reduced bacterial burden in murine models without impacting bacterial growth, a critical advantage in minimizing resistance development [202].

Clinical trials are now warranted to assess the safety and efficacy of promising candidates. Topical agents such as *S. warneri* AIPs, which demonstrated dose-dependent *agr* suppression and reduced MRSA-induced skin damage in vivo [164], merit phase I safety evaluations. Implant coatings, including MPDA-LUT@CaP, which achieved 90.3% biofilm elimination in osseointegration models [224], necessitate testing in orthopedic implant contexts. Repurposed drugs like sitagliptin, which protected mice from *S. aureus* and *P. aeruginosa* co-infections [183], warrant evaluation in high-risk cohorts such as diabetic foot ulcers.

Future research should focus on optimizing pharmacokinetics for compounds with dosing limitations. For example, MA01 rhamnolipid’s high effective concentrations [178] and resveratrol’s sub-inhibitory thresholds [207] necessitate formulation enhancement. The role of host-microbe interactions must be further explored; probiotic metabolites have shown the potential to increase oxidative stress susceptibility in *S. aureus* [179], indicating the value of symbiotic approaches in mucosal infections. Resistance monitoring is also essential—longitudinal studies for agents like Syph-1, which thus far show no resistance induction [194], are crucial to validate their long-term durability.

These efforts are key to bridging the gap between preclinical promise and clinical applicability, ensuring that anti-virulence therapies are equipped to address the adaptive complexity of *S. aureus* infections.

## 8. Strategies to Prevent Resistance in Anti-Virulence Interventions

This review consolidates recent advances in anti-virulence therapeutics targeting *S. aureus* and other pathogens, focusing on strategies that disrupt virulence pathways without exerting bactericidal pressure. Key innovations include, but are not limited to, the following:

1. Multi-target and dual-action mechanisms: Several compounds, such as MPDA-LUT@CaP implant coating [224] and HSGN-220/HSGN-218 [155], synergistically inhibit QS and biofilm formation while disrupting bacterial metabolism (e.g., iron starvation, DNA replication). These multi-pathway approaches reduce the likelihood of resistance by limiting adaptive escape routes for pathogens.
2. Non-bactericidal virulence suppression: Agents like *S. simulans* AIP-I [163] and macrocyclic QS quencher peptides [185] selectively block QS receptors (e.g., AgrC) or virulence regulators (e.g., BfmR) at sub-inhibitory concentrations, attenuating pathogenicity without affecting bacterial viability. This minimizes selective pressure for resistance.
3. Repurposed drugs with dual utility: Bumetanide [196] and fenoprofen [213], originally developed for non-infectious conditions, inhibit AgrA and SaeR signaling, respectively. They demonstrate efficacy in vivo without inducing resistance. Their established safety profiles expedite clinical translation.
4. Natural product-derived solutions: Compounds like propolis triterpenoids [193] and rosemary extracts [165] leverage natural QS inhibitors with multi-target activity, offering ecological compatibility and reduced resistance risk.

To advance these concepts, the authors propose the following:

- Optimization of bioavailability: Addressing limitations like poor solubility (e.g., physalins; [173]) or high effective concentrations (e.g., MA01 rhamnolipid; [178]) through formulation technologies (e.g., nanoparticle encapsulation).
- In vivo validation of multi-target agents: Prioritizing preclinical studies for compounds like synthetic gallotannins [153] to confirm their resistance-sparing potential in complex host environments.
- Exploration of host–pathogen interface: Leveraging immunomodulatory agents (e.g., Saikosaponin A; [157]) that enhance host defenses while indirectly suppressing virulence, thereby avoiding direct microbial targeting.

The review underscores a paradigm shift toward anti-virulence therapeutics that decouple efficacy from resistance selection by focusing on these non-lethal, multi-mechanistic strategies. This approach aligns with global efforts to combat antimicrobial resistance while expanding the arsenal against infections that are resistant to treatment.

## 9. Limitations and Challenges

Anti-virulence strategies against *S. aureus* hold great promise by disarming pathogenic mechanisms without exerting direct bactericidal pressure. However, they face several notable constraints that hinder their successful translation into clinical practice. One major issue is the highly variable efficacy of these agents against different *S. aureus* strains, reflecting the bacterium’s extensive genetic diversity. Strain-specific differences in virulence factors, regulatory elements, and biofilm composition can limit the broad-spectrum applicability of many anti-virulence molecules. Further compounding this problem is the structural resilience of biofilms, whose extracellular matrix protects the bacteria from host immune responses and therapeutic interventions. As a result, agents that effectively reduce biofilm formation in vitro may still struggle to eradicate mature biofilms or achieve consistent in vivo results.

Another significant challenge lies in these compounds’ pharmacokinetic and formulation hurdles. Many of them exhibit suboptimal stability, poor bioavailability, or extensive degradation within the host, making it difficult to maintain therapeutic concentrations at infection sites. Additionally, the molecular mechanisms by which specific agents inhibit virulence factors or disrupt regulatory pathways are not fully elucidated, complicating rational drug design and optimization. Prolonged exposure to these agents also carries the risk of selective resistance, especially if the anti-virulence mechanism inadvertently favors the survival of strains with altered regulatory circuits. Finally, translating promising in vitro findings into clinical efficacy requires navigating host–pathogen interactions, immune responses, and microbiome dynamics that can significantly alter therapeutic performance. These overlapping limitations and challenges underscore the multifaceted nature of anti-virulence therapy and highlight the necessity for integrative research that addresses variability in bacterial strains, refines pharmacological properties, and evaluates long-term safety.

## 10. Future Directions

Advancing anti-virulence strategies against *Staphylococcus aureus* requires targeted innovations in drug design, biofilm disruption, and clinical translation.

A key priority is optimizing small-molecule inhibitors and peptide-based therapeutics targeting adhesion proteins (e.g., ClfA and ClfB) and QS regulators (e.g., AgrA and SarA). High-throughput screening and structural refinements can enhance specificity and minimize host interactions. Combination approaches that integrate anti-virulence agents with antibiotics or antimicrobial peptides can enhance therapeutic efficacy while reducing the selective pressure for resistance.

Biofilm resilience remains a significant challenge, necessitating advanced delivery systems such as nanoformulations and bacteriophage-derived therapies to enhance drug penetration and stability. Functionalized medical implants and surface-modifying agents hold promise for preventing biofilm formation in clinical settings.

Translational efforts must prioritize in vivo validation and clinical trials to assess the pharmacokinetics, safety, and efficacy. Additionally, real-time genomic surveillance will be critical in monitoring bacterial adaptation and resistance evolution. By integrating these approaches, anti-virulence strategies could emerge as viable adjuncts to conventional antibiotics in combating persistent *S. aureus* infections.

## 11. Conclusions

This review highlights the promise of anti-virulence strategies to mitigate *Staphylococcus aureus* infections by reducing pathogenicity without applying direct bactericidal pressure. Nevertheless, challenges remain, including strain variability, biofilm resistance, and pharmacokinetic issues, as well as risks of resistance. We recommend combining anti-virulence therapies with antibiotics, developing improved formulations, and focusing on translational studies that integrate genomic, proteomic, and metabolomic data, alongside investigations into host–pathogen-microbiome interactions for clinical validation.

## Supplementary Materials

The following supporting information can be downloaded at: https://www.mdpi.com/article/10.3390/pathogens14040386/s1, Table S1: Comprehensive summary of antivirulence factors: efficacy, advantages, and limitations against *Staphylococcus aureus*.

## Abbreviations

The following abbreviations are used in this manuscript:

10-HDA	10-Hydroxy-2-decenoic Acid
3,4′-DMF	3,4′-Dimethoxyflavone
Agr	Accessory Gene Regulator
AgrA	Agr Response Regulator
AgrC	Agr Sensor Kinase
AIP	Autoinducing Peptide
ArlRS	Arl Two-Component System
bCSE	Bacterial Cystathionine-γ-Lyase
Clfs	Clumping Factors
ClpP	Caseinolytic Protease
CrtM	Dehydrosqualene Synthase
CrtN	Dehydrosqualene Desaturase
DSS	Dextran Sodium Sulfate
EGCG	Epigallocatechin Gallate
EPS	Extracellular Polymeric Substances
ESKAPE	*Enterococcus faecium*, *Staphylococcus aureus*, *Klebsiella pneumoniae*, *Acinetobacter baumannii*, *Pseudomonas aeruginosa*, *Enterobacter* species
FeoB	Ferrous Iron Transporter
FnBPs	Fibronectin-Binding Proteins
GraXRS	GraXRS Two-Component System
H2S	Hydrogen Sulfide
Hla	Alpha-Hemolysin
HPC	Homopterocarpin
HXS	Hexestrol
IsdB	Iron-Regulated Surface Determinant B
KD	Dissociation Constant
LL-37	Human Cathelicidin LL-37
mAbs	Monoclonal Antibodies
MK-4	Menaquinone-4
MRSA	Methicillin-Resistant *Staphylococcus aureus*
NDH-2	Type II NADH Dehydrogenase
PBP2a	Penicillin-Binding Protein 2a
PP-HCl	Phenazopyridine Hydrochloride
PSMs	Phenol-Soluble Modulins
PVL	Panton–Valentine Leukocidin
QS	Quorum Sensing
*S. aureus*	*Staphylococcus aureus*
SaeRS	Sae Two-Component System
SarA	Staphylococcal Accessory Regulator A
SCCmec	Staphylococcal Chromosomal Cassette mec
SrtA	Sortase A
STX	Staphyloxanthin
TCS	Two-Component Systems
TSST-1	Toxic Shock Syndrome Toxin-1
VRSA	Vancomycin-Resistant *Staphylococcus aureus*
YycFG	YycFG Two-Component System
