# Supplementary material for: Disarming Staphylococcus aureus: Review of Strategies Combating This Resilient Pathogen by Targeting Its Virulence
Source: Pathogens. 2025 Apr 15;14(4):386. doi: 10.3390/pathogens14040386 (PMC12030135; doi:10.3390/pathogens14040386)
Supplement: Supplementary file 1 [file pathogens-14-00386-s001.zip › pathogens-3544246-supplementary.pdf]

Supplementary Table S1: Comprehensive summary of antivirulence factors: efficacy, advantages, and limitations against *Staphylococcus aureus*

Antivirulence Factor	Efficacy	Advantage	Disadvantages
**10-Hydroxy-2-decenoic acid**	41.9–72.9% biofilm biomass reduction; inhibits α-hemolysin	Natural compound; dual anti-biofilm and anti-virulence	Sub-MICs required; limited direct bactericidal activity
**2B4 receptor blockade**	Reduces SEB-induced eosinophil activation and inflammation	Novel target; mitigates allergic inflammation	Specific to SEB-2B4 interaction; untested in humans
**2R,3R-Dihydromyricetin**	IC50: 73.43 μM (SrtA); reduces adhesion by 1.14-1.75 log CFU/cm^2	Safe for food applications; binds SrtA catalytic pocket	Moderate potency compared to other SrtA inhibitors
**3,2'-Dihydroxyflavone**	MIC 75 μg/mL; inhibits polymicrobial biofilms	Targets dual-species infections	Not specified
**3,4'-DMF, HPC**	Reduced MRSA virulence in murine skin infection; prevented dermonecrosis.	Targets quorum sensing without affecting bacterial growth.	Mechanism of HPC not fully elucidated.
**5-Fluorouracil**	Reduces AI-2; enhances daptomycin efficacy *in vivo*.	Synergizes with antibiotics; prevents resistance.	Potential off-target effects (originally an oncology drug).
**7,8-Dihydroxyflavone**	32 μg/mL inhibits Hla; synergizes with vancomycin *in vivo*	No host cytotoxicity	Not specified
**Actinomycetales metabolites**	Phenalinolactones A–D inhibit AgrA ATP site; suppress hemolysis.	Dual *agr* and *fsr* inhibition (Synerazol).	Synergistic effects with antibiotics not tested.
**Ag@Glu/Tsc Nanoparticles**	Reduces biofilm biomass by 76.7%; downregulates *icaA/icaD* by 60-67%	Functionalized for targeted delivery; induces oxidative stress in biofilms	Risk of silver resistance; nanoparticle aggregation in biological environments
**Ag-Au-Pd Nanoapatites**	MIC: 128 μg/mL; inhibits MRSA/VRE biofilms	Low cytotoxicity; promotes fibroblast survival in biofilm-infected conditions	Complex synthesis process; potential long-term biocompatibility concerns
**AgNPs (*H. speciosa*)**	92.41% biofilm eradication (0–25 μg/mL)	Green synthesis; broad anti-biofilm activity	Potential cytotoxicity of silver nanoparticles

**Allantodapsone**	IC50: 21.3 mM (fibrinogen); inhibits ClfA/ClfB	First MSCRAMM inhibitor; prevents colonization	High IC50 may limit therapeutic utility
**Ambuic acid**	Suppress *agr* and *fsr* systems; reduce hemolysin and biofilm.	Broad-spectrum QS inhibition.	Epoxide reactivity may cause off-target effects.
**AMPT (Thiazole Derivative)**	MIC lower than vancomycin; inhibits biofilm and virulence.	Superior bactericidal activity; targets AgrA/SarA.	Stability and delivery challenges.
**Anandamide**	Reduces SEB-induced ARDS via miRNA modulation and Treg/MDSC expansion	Immunomodulatory; natural endocannabinoid	Psychoactive potential (if THC-derived); route-specific efficacy
**Anti-SraP L-lectin mAb**	Reduces bacterial load in bloodstream; blocks adhesion	Targets key adhesin; passive immunization strategy	Requires antibody administration; no active immunity
**Apigenin-7-O-glucoside**	MBIC 0.20 mg/mL; reduces biofilm by 88.9%.	Natural compound; reduces hydrophobicity and EPS.	Limited to *S. aureus* and *E. coli*.
***Artemisia dracunculus* EOs**	MIC 1.25 µL/mL; inhibits biofilm and QS genes.	Natural source; disrupts pre-formed biofilms.	Estragole (main compound) may have toxicity concerns.
**Artesunate**	Sub-MIC reduces adhesion and biofilm	Targets quorum sensing	Not specified
**Avacopan**	Reduces PVL EC50, suppresses IL-1β	Repurposed drug; adjunctive therapy	Some compounds (e.g., BM213) enhance cytotoxicity
**Ayanin**	Inhibits ClpP (IC50: 19.63 µM); reduces virulence factors (e.g., *hla*)	Synergizes with vancomycin; targets multiple virulence pathways	Moderate IC50
**Azan-7**	Inhibits AgrA; enhances clindamycin susceptibility.	No resistance development; stable long-term.	Requires adjunct therapy; bioavailability issues.
**Azithromycin**	Reduces hemolytic activity at sub-MIC	Reduces inflammatory damage (e.g., ocular)	Mechanism not fully elucidated
***B. subtilis* Peptides**	Disrupts biofilms across *agr* types; enhances antibiotic susceptibility.	Probiotic-derived; protects host cells.	Unidentified peptide components; requires formulation.
**Baicalein**	Inhibits vWbp; improves survival in pneumonia model	Enhances penicillin G efficacy	Herbal extraction complexity

**Bicyclomycin**	Increased SaeRS-dependent virulence factors.	N/A (study highlights risk).	Paradoxically enhances virulence; not recommended.
**Biochanin A**	32 μg/mL inhibits Hla; reduces lung bacterial load	Improves survival in lethal MRSA model	Not specified
**Bisdemethoxycurcumin**	Downregulates agrA; inhibits biofilm formation	Natural compound, targets quorum sensing	Not specified
**Bispecific scFV antibody (MB102a)**	Allosterically blocks SEB-TCR binding; prevents immune activation	Dual targeting; computationally optimized	Complex production; stability *in vivo* uncertain
**BLS P34**	Reduces *S. aureus* biofilm metabolism by 41-95%	Food-safe bacteriocin-like peptide	Ineffective against preformed biofilms; stimulates *E. faecalis* metabolism
**Bumetanide**	70% AgrA inhibition at 0.1 μM; reduces ulceration *in vivo*.	Repurposed drug; no resistance or toxicity observed.	Off-target diuretic effects in systemic use.
**C1/C2 (*L. plantarum*)**	Reduces biofilm formation; downregulates *icaC/D*	Novel compounds; effective against MRSA	Uncharacterized long-term safety
**Candesartan, Domperidone, Miconazole**	Sub-MIC inhibition of virulence factors; candesartan downregulates icaA, hla, crtM	FDA-approved, reduces resistance pressure	Not specified
**Carboxypyranoanthocyanins**	Reduces *S. aureus* biofilm; downregulates *agrA*.	Non-toxic; synergizes with antibiotics.	Ineffective against *P. aeruginosa* biofilm.
**Celastrol**	Inhibits CrtM; reduces STX and biofilm	Multi-targeted; enhances oxidative stress susceptibility	Potential toxicity from plant source (*Tripterygium*)
**Chitosan-coated Surfaces**	Reduces *S. aureus* biofilm by 79%	Biocompatible; reduces viable but nonculturable (VBNC) cells	Lower efficacy against *P. aeruginosa* (66%)
**Chlorothymol**	MIC: 32 μg/ml; inhibits biofilm/motility at 8 μg/ml	Synergizes with oxacillin (FIC: 0.3125); membrane disruption	High MIC for clinical use
***Chrysanthemum* EOs**	Inhibits MRSA growth, biofilm, and virulence genes (mecA, sea, agrA)	Natural antimicrobial; combats antibiotic resistance	Concentration-dependent efficacy; unstandardized extracts

**CHX-loaded nanoHA**	Dose-dependent bacterial inhibition; biocompatible at low doses	Controlled release; medical device coating	Cytotoxic at high CHX concentrations
**Citral**	MIC 5–40 mg/mL; reduces early-stage biofilm	Adjunct to conventional therapies	High concentrations required
**Compound 5, N1, N3, N9, N10 (VraSR inhibitor)**	Inhibited VraS ATP-binding (ΔGbind -294.32 kJ/mol); disrupted VraR-DNA binding.	Reduces antibiotic resistance mechanisms.	Requires validation in clinical isolates.
**Coptisine**	Inhibits SrtB; protects lung epithelial cells	Avoids antibiotic resistance; targets adhesion	Indirect mechanism; no direct antibacterial activity
**Coumarin-chalcone conjugate**	Reduces biofilm biomass (30%); downregulates *lasI/R*, *rhlI/A*.	Broad-spectrum QS inhibition; sensitizes biofilms to antibiotics.	Mechanism of cyclic-di-GMP modulation unclear.
**Daphnetin**	IC50: 25.98 µg/mL (SrtA); suppresses hemolysis and motility	Dual targeting of SrtA and α-hemolysin; protects against pneumonia *in vivo*	Binding affinity may vary across strains
**D-Asp/D-Glu + Ciprofloxacin**	97% biofilm inhibition/dispersal at 40 mM	Disrupts eDNA; enhances antibiotic efficacy	High amino acid concentration required
**Delta-9-THC**	Prevents SEB-induced ARDS mortality; suppresses cytokines via CB2 receptor	Effective post-exposure; promotes anti-inflammatory response	Psychoactive side effects; regulatory hurdles
**Diclofenac, Meloxicam**	47–59 µg/mL inhibits STX by 79–98%	Binds CrtM, adjuvant potential	Not specified
**DLL37-1/LL37-1**	52-58% biofilm inhibition at 5 µM	No hemolysis/cytotoxicity up to 5 µM; targets MSCRAMM proteins	Limited data on *in vivo* efficacy
**DPTM (pleuromutilin derivative)**	Dose-dependent Hla/agrA downregulation; reduces inflammation	Anti-inflammatory, protects host cells	Not specified
**D-Serine**	Reduces MRSA adhesion and biofilm genes (*agrA*, *sarS*)	Non-antibiotic; specific to biofilm inhibition	Efficacy in complex *in vivo* models requires validation

**DSS (SaeR inhibitors)**	Inhibited biofilm attachment via secreted protein.	Species-specific biofilm control.	Inhibitory protein identity remains unknown.
**DSS and I-modulia®**	Suppressed δ-toxin and SaeRS-regulated genes.	Synergistic effect; safe for skin microbiota.	Limited to topical applications (e.g., dermatitis).
**EGCG and NOL polyphenols**	Suppress SEA production and biofilm-related genes in MVs	Natural compounds; dual anti-inflammatory and antibiofilm effects	Variable potency (EGCG > NOL); limited *in vivo* data
**EGCG-functionalized p(HEMA-co-GMA)**	71% reduction in *S. aureus* adhesion (1000 µg/mL, 4h)	Prevents contamination in biomedical devices and food packaging	Dose-dependent efficacy; high concentrations required
**Engineered chromones**	Inhibit TSST-1 via high-affinity binding; optimized drug-like properties	Targets antibiotic-resistant strains; good solubility and permeation	Early-stage design; untested in animal models
**Ensifentrine**	Preserves lung barrier function; reduces IL-6/IL-8 and endothelial leakage	Mitigates MRSA-induced ARDS; EPAC-mediated anti-inflammatory effects	Does not directly target bacterial viability
**Eugenol**	Inhibits AgrA phosphorylation; reduces ATP and enterotoxin production.	Natural compound; effective in food safety applications.	High concentrations may affect host cells.
**Fenoprofen**	Attenuated biofilm pathogenicity in implant models.	FDA-approved repurposing; no resistance induction.	NSAID side effects in chronic use.
**Flavone derivatives**	Reduced oxacillin resistance in MRSA.	Dual antivirulence and resistance modulation.	Mechanism via GraRS or mecA-PBP2a remains unclear.
**Flavuside B**	Suppresses *agrA* (1.7-fold); promotes wound healing.	Dual antioxidant and anti-QS effects; in vitro wound model.	Marine-derived; scalability challenges.
**Fmoc-F**	Inhibits biofilm ECM components; synergizes with antibiotics	Effective as a coating material; targets multiple ECM components	Mechanism of ECM interaction not fully elucidated
**Forsythiaside**	Inhibits adhesion (>30 mg/mL); attenuates NF-κB	Dual antibacterial and anti-inflammatory activity	High concentrations needed for biofilm prevention
**G5-QQ3 Dendrimer**	Biofilm inhibition (60–72%); MIC_{50} 18 µM.	Enhanced peptide stability; penetrates biofilms.	Complex synthesis; untested *in vivo*.

**GA, NGA (SaeR inhibitors)**	Inhibited biofilm formation and virulence gene expression in vitro and *in vivo*.	Dual activity against planktonic and biofilm-associated MRSA.	Potential off-target effects due to broad transcriptomic changes.
**Galangin**	Inhibits vWbp; reduces bacterial load *in vivo*	Enhances latamoxef; binds directly to vWbp	Mechanism specificity
**Gallic Acid**	MIC: 32 μg/mL; inhibits biofilms at 8 μg/mL	Reduces PIA production; downregulates *sarA/icaA/icaD*	Limited efficacy against mature biofilms at low concentrations
**GW3965·HCl (FeoB Inhibition)**	Reduces FeoB activity, staphyloxanthin, and bacterial growth	Synergistic with host oxidative stress; low resistance development	Specificity and pharmacokinetics not fully characterized
**Hexestrol**	MIC: 16 μg/mL; reduces EPS and biofilm-related genes	Synergistic with aminoglycosides; targets MRSA	Synthetic estrogen may have off-target effects
**Hibifolin**	IC50: 31.20 mg/mL for SrtA; enhances cefotaxime efficacy	Adjuvant therapy; reduces antibiotic resistance risk	High IC50 limits standalone use
**HR3744, SAV13 (SaeR inhibitors)**	Reduced α-toxin and PVL expression; effective in murine models.	High specificity; no selective pressure.	SAV13 potency requires toxicity evaluation.
**HSGN-220, HSGN-218**	MIC = 0.06–0.5 μg/mL against MRSA; disrupts DNA replication, iron starvation, and membrane potential	Multi-target action; low resistance propensity	Complex synthesis; potential off-target effects in hosts
**Ibuprofen**	Synergy with vancomycin enhances wound healing in rats	Enhances traditional antibiotics	Not specified
**Iclaprim**	Strain-dependent Hla reduction; depolymerizes biofilms at 1 MIC	Improves survival in *Galleria* model	Strain-dependent efficacy
**Isoquercitrin**	Inhibits Coa (binds Asp-181/Tyr-188); reduces bacterial load *in vivo*	Improves survival in pneumonia; non-bactericidal	Limited to Coa-targeted therapy
**Isorhapontigenin**	Inhibits MgrA; synergizes with vancomycin in murine pneumonia	Reduces virulence gene expression; enhances immune response	No direct bactericidal activity
**Isosakuranetin**	IC50: 21.20 μg/mL for SrtA; reduces lung damage *in vivo*	Dual inhibition of SrtA and Hla; anti-inflammatory	Low IC50 requires further validation

**Isovitexin**	Inhibits Coa activity; prevents fibrin formation	No impact on bacterial growth	Specific to Coa; limited broad-spectrum applicability
**Iturins (*Bacillus velezensis*)**	76% hemolysis reduction	No resistance pressure, livestock applications	Not specified
**Kaempferol**	32 μg/mL inhibits Hla; improves survival in pneumonia model	Protects lung tissue	Not specified
***L. helveticus* Biosurfactants**	Inhibits quorum sensing via AI-2; reduces *icaA/sarA/agrA* expression	Safe, natural alternative; prevents host cell invasion	Strain-dependent efficacy (e.g., 27170 > 27058)
***Lactobacillus* strains**	Moderate inhibition of *S. aureus* adhesion	Safe for food/pharmaceutical use; natural origin	Variable strain efficacy; live cells required for optimal effect
**Lignin-capped AgNPs**	MIC 10 μg/mL; downregulates *luxR*, biofilm genes.	Non-toxic to human cells; QS and biofilm dual targeting.	Risk of metal resistance gene upregulation.
**LL-37**	MIC: 0.62 mM; reduces adhesion at ≥0.16 mM; disrupts biofilms at 5 mM	Potent anti-biofilm activity; targets titanium implant infections	High concentration required for mature biofilms
**Lysine**	Reduces hemolysis; protects intestinal mucosa in mice	Food-safe, biocompatible	Not specified
**MA01 rhamnolipid**	70% reduction in MRSA viability; inhibits biofilm via *agrA*, *agrC*, *icaA/D* downregulation.	Natural biosurfactant; dual QS and biofilm inhibition.	High effective concentrations (30–120 mg/mL).
**mAbs (Hla, Luk, ClfA)**	69% survival improvement in septic shock model	Multi-toxin targeting	Not specified
**Macrocyclic QQ peptides**	IC_{50} ~1 nM; blocks AgrC activation.	High affinity; resistance unlikely due to non-bactericidal action.	Peptide stability and delivery challenges.
**Maleimide-diselenide**	Inhibits biofilm via *sarX* suppression; reduces nasal colonization	Low resistance risk; *in vivo* efficacy	Early-stage research; limited toxicity data
**MAS-19/MAS-30 (phenyl esters)**	>60% inhibition of ClpXP; reduces hemolysis	High stability, minimal cytotoxicity	Not specified
**Metal Oxide Nanoparticles**	Biofilm disruption (80–88%); MIC 0.63 mg/mL (ZnO).	Broad-spectrum; durable.	No QS inhibition; bactericidal mechanism only.

**Microalgal EPS (*T. suecica*)**	Reduces adhesion via surface hydrophilicity	Sustainable; non-toxic coating for food surfaces	Limited to specific surfaces (e.g., polystyrene)
**Monoclonal antibody Hm0487**	Neutralizes SEB by blocking TCR/MHC-II interaction; improves survival in mice	High affinity; targets novel epitope distant from TCR/MHC-II sites	Requires cold storage; high production costs
**Monoclonal antibody LXY8**	Neutralizes SEB with 0.525 nM affinity; protects against toxic shock	High potency; human-derived for reduced immunogenicity	Requires parenteral administration; costly production
**Monoclonal antibody YG1**	Neutralizes Hla; protects against bacteremia/pneumonia	Specific epitope targeting	Not specified
**Morin**	Inhibits biofilm (sub-MIC); disrupts EPS production.	Non-bactericidal (reduces resistance risk); targets SarA protein.	Negligible direct antibacterial activity.
**MPDA-LUT@CaP implant coating**	95.59% antibacterial rate; 90.3% biofilm elimination.	Dual PTT-QSI action; promotes osseointegration.	Requires NIR activation; acidic microenvironment dependency.
**mRNA-based SEB vaccines/mAbs**	Induces robust immune responses; superior to protein-based vaccines	Rapid development; long-lasting immunity	Requires cold chain storage; potential mRNA instability
**MSI-1 peptide**	Bacteriostatic/bactericidal; inhibits STX, synergizes with vancomycin	Targets LTA and CrtN; effective against MRSA/VRSA	Toxicity not fully assessed
**Multicomponent toxoid vaccine (IBT-V02)**	Generates neutralizing antibodies against Hla, PVL, LukAB, SEB, SEA, TSST-1	Broad protection; effective in pre-exposed hosts	Complex formulation; potential antigen competition
**Myricetin**	Reduces lung injury in mice; inhibits biofilm	Dietary flavonoid, adjunctive therapy	Not specified
**Naphto-γ-pyrones (NGPs)**	Inhibits biofilm/hemolysis (80%); enhances vancomycin efficacy	Non-bactericidal; marine fungal origin	Production scalability from marine fungi
**Naringenin (Hass avocado extract)**	Binds SElX; inhibits MRSA biofilm and toxin production	Natural compound; dual antimicrobial and antivirulence effects	Variable bioavailability; unoptimized dosing
**NH125 (VraSR inhibitor)**	Sensitized MRSA to carbenicillin/vancomycin.	Noncompetitive kinase inhibition; combinatorial potential.	High concentrations required for efficacy.

**Non-digestible oligosaccharides**	Inhibit bacterial adherence, biofilm formation, and toxin receptor mimicry	Broad-spectrum; prebiotic benefits for gut microbiota	Efficacy dependent on gut microbiota composition
**Norlichexanthone**	Reduced toxins and biofilm formation.	Dual targeting of Agr and SaeRS.	Fungal-derived compound; scalability concerns.
**Nusbiarylins**	Reduces toxin production at sub-MIC	Targets transcription, avoids resistance	Not specified
**PASP and EO@PASP/HACCNPs**	1.20–1.68 log CFU/mL biofilm reduction; 97.35% inhibition rate	Enhances food safety; preserves sensory properties	Complex nanoparticle synthesis
**Patuletin**	Reduces biofilm (27-23%) and staphyloxanthin (53-46%) at 1/4 MIC	Synergizes with antibiotics; stable CrtM binding	Sub-inhibitory concentrations may not kill bacteria
**PEINF Nanoparticles**	Inhibits biofilm and motility; QS inhibition in *C. violaceum*.	Broad activity (bacteria and fungi); durable effects.	No direct QS inhibition in *S. aureus*.
**Petroselinic acid**	Inhibits biofilm on abiotic/skin surfaces; downregulates agrA, RNAIII	Non-toxic, targets quorum sensing	Not specified
**Phenazopyridine HCl**	Inhibits TSST-1 via SaeRS TCS suppression; spares microbiota	Targets virulence without affecting growth; preserves vaginal microbiota	Narrow focus on TSST-1; unconfirmed efficacy against other toxins
**Photoactivated Ga^{3+}CHP**	Reduces SEC/TSST-1 production via ROS generation; lowers cytotoxicity	Non-antibiotic; targets skin colonization; safer for human cells	Requires light activation; limited to topical applications
**PhPr(3Br)-Bnc3 peptidomimetics**	Sub-nanomolar IC_{50} across all *agr* groups.	Pan-*agr* inhibition; structural tunability.	Synthetic complexity and cost.
**PHT-427 (FeoB Inhibition)**	Reduces staphyloxanthin, biofilm, and enhances antibiotic susceptibility	Safe in animal models; broad Gram-positive activity	Limited to bovine mastitis model; no human trial data
**Physalins**	Block AgrA-DNA binding; reduce hemolytic toxin production.	Plant-derived; target DNA-binding site.	Limited solubility or bioavailability data.
**Piceatannol**	Binds Hla; disrupts pore formation	Direct toxin interaction	Not specified

**Plantamajoside**	IC50: 22.93 µg/mL (SrtA); synergizes with vancomycin	Reduces immune evasion; effective in murine and *Galleria* models	Requires combination therapy for optimal MRSA eradication
**PMI-5**	Reduced hemolysis by 65%; decreased lesion size in murine models.	Broad inhibition of virulence-associated TCSs.	Requires further pharmacokinetic studies.
**Pomegranate/persimmon extracts**	PoPE reduces QS pigments; GrPE disrupts biofilms.	Food waste utilization; multi-pathogen activity.	Variable efficacy across extracts.
**PP-HCl (SaeR inhibitors)**	Downregulated TSST-1; mitigated T-cell activation.	Preserves vaginal microbiota; non-bactericidal.	Specific to toxin-mediated infections (e.g., menstrual TSS).
**Probiotic Metabolites**	Downregulates *agrA*; enhances oxidative stress susceptibility.	Non-antibiotic strategy; safe for host.	Strain-specific effects; requires co-administration.
**Propolis Triterpenoids**	18–40% biofilm inhibition at MIC.	Natural product; targets QS and virulence.	Moderate efficacy; limited to *S. aureus*.
**Pyocyanin**	MIC 8 µg/mL; eradicates 83–88% biofilms.	Targets AgrA; inhibits motility and virulence.	Potential cytotoxicity at higher doses.
**Pyrazolopyrimidine**	Suppresses PSMs; no growth inhibition.	High-throughput screening validated; specific *agr* targeting.	Limited *in vivo* efficacy data.
**Quebrachitol**	Inhibits biofilm without affecting growth; downregulates *sarA/agr/ica*	Broad-spectrum anti-adhesive activity; natural compound	No direct bactericidal effect
**Quercetin**	Prevents αHL-induced hemolysis at sub-MIC	Protects host membranes	Does not inhibit toxin production
**Quercetin + antibiotics**	Enhances ROS production; disrupts biofilm	Broad-spectrum, amplifies antibiotic effects	Host membrane modulation (indirect)
**Resveratrol**	Binds AgrC (−8.9 kcal/mol); stable in MD simulations.	Superior pharmacokinetics vs. penicillin; natural origin.	Limited *in vivo* validation.
**Rhodionin**	IC50: 22.85 µg/mL for SrtA; improves survival in pneumonia model	Targets virulence without affecting growth; *in vivo* efficacy	Moderate potency
**Robusta Coffee Extracts**	39.69% biofilm inhibition at ½ MIC.	Natural source; inhibits mature biofilms.	Moderate activity; limited to in vitro models.

**Rosemary extracts (carnosic acid)**	Inhibits *agr* at 5 μM; reduces PSMs and α-hemolysin.	Natural source; anti-inflammatory potential.	Variable potency between compounds (carnosol vs. rosmarinic acid).
**Rosemary/Myrtle EOs**	Reduces biofilm (74%); inhibits hemolysin/DNase	Natural combination; ROS-mediated oxidative stress	Variable MICs (0.7–11.25 mg/mL)
**Rutin-loaded chitosan NPs**	Reduces staphyloxanthin; inhibits biofilm (22.5-37.5%)	Multi-mechanistic; enhances H2O2 susceptibility	Nanoparticle delivery challenges
***S. simulans* AIP-I**	Nanomolar inhibition of all MRSA *agr* types; reduces dermonecrosis *in vivo*.	Broad-spectrum *agr* suppression; non-antibiotic.	Limited clinical validation.
***S. warneri* AIPs**	Dose-dependent *agr* inhibition; reduces MRSA skin damage *in vivo*.	Commensal-derived; topical applicability.	Variability in AIP potency (AIP-II > AIP-I).
**S-342-3**	Reduces biofilm mass by 25-57% at 4 μg/mL	Non-toxic to human cells and *Galleria*; downregulates *agrA/sarA*	Sub-MIC use limits bactericidal activity
**Saikosaponin A**	Reduces TNF-α, IL-1β, and ferroptosis markers (e.g., MDA, GPX4)	Modulates host inflammation and iron metabolism	Indirect antibacterial effect; requires host cooperation
**scFv MS473**	100% survival in TSST-1-induced shock; prevents organ damage	Fully human; effective post-exposure therapy	Limited to TSST-1; requires early administration
**SEA-specific aptamer (Apt5)**	Detects SEA at 100 ng (food poisoning threshold)	Rapid lateral flow assay compatibility; no antibodies required	Limited to diagnostic applications
**SEB mimetic peptide (pSEB116-132)**	Blocks SEB-CD28 binding, reduces cytokine production, restores barrier integrity	Specific targeting; mitigates intestinal dysfunction	Limited to SEB; potential delivery challenges
**SEB-targeting nanobody (Nb8)**	Inhibits SEB-MHC-II interaction; blocks inflammatory responses	Small size; high stability; engineered for specificity	Potential immunogenicity; unproven in human trials
**SED-specific aptamer (Aptamer 1)**	Detects SED with high sensitivity (LOD: 45 nM)	Cost-effective; reproducible alternative to antibodies	Diagnostic use only; limited therapeutic application

**Shikonin**	Downregulates *agrA*, *RNAIII*; 70% biofilm inhibition; promotes wound healing.	Dual antimicrobial and tissue-regenerating effects.	Polymicrobial biofilm efficacy requires further validation.
**Short-chain fatty acids (SCFAs)**	Modulate intestinal environment; inhibit pathogen growth and toxin activity	Naturally derived; enhances commensal microbiota	Variable concentration in gut; indirect action
**Sidr Honey**	MIC 50–400 mg/mL; inhibits biofilm (70.88%).	Broad-spectrum; antioxidant properties.	High MIC limits practical use.
**Silver nanoparticles**	Downregulates *ica*, *agr*, and virulence genes	Broad-spectrum; disrupts biofilm architecture	Risk of silver resistance; cytotoxic at high doses
**Sinensetin**	Inhibits Coa (128 μg/mL); reduces biofilm	*In vivo* efficacy; synergizes with oxacillin	High concentration required
**Siphonocholin**	60% biofilm inhibition; binds BfmR QS regulator.	Marine-derived; dual QS and biofilm suppression.	Limited mechanistic studies in MRSA.
**Sitagliptin**	Inhibits biofilm (both *S. aureus* and *P. aeruginosa*); protects mice.	Repurposed drug with known safety; downregulates multiple QS genes.	Limited to specific QS pathways; requires clinical validation.
**SKKUCS (SaeR inhibitors)**	Reduced bacterial burden in murine models; no growth impact.	High-throughput validated; low resistance risk.	Mechanism limited to kinase ATP-binding site.
**Snail mucus fractions**	MIC20 reduces virulence factors	Natural source, antibiofilm activity	Source availability challenges
**Staquorsin**	Inhibits AgrA binding; reduces RNA III, hemolysins, lipases; *in vivo* reduction of bacterial burden.	Minimal impact on viability; no resistance observed.	Long-term safety profile not fully established.
**Sumra Honey**	Inhibits biofilm (48%) and QS (68.7%).	Rich in bioactive compounds; natural.	High MIC (300 mg/mL); impractical for clinical use.
**Surfactin**	Inhibits biofilm formation on multiple surfaces	Natural biosurfactant; modulates quorum sensing	Stability in practical applications unclear
**SYG-180-2-2**	Reduces abscess formation in mice; sensitizes to oxidative stress	No growth inhibition, *in vivo* efficacy	Not specified
**Synthetic Gallotannins**	Sub-MIC biofilm inhibition; disrupts quorum sensing	Effective against MRSA biofilms; antioxidant properties	Moderate direct antibacterial activity (MICs not specified)

**Tamarixetin**	Inhibits ClpP (IC50: 49.73 μM); suppresses *hla*, *agr* transcription	Non-cytotoxic; enhances cefotaxime efficacy	Higher IC50 compared to ayanin
**Terbinafine**	Inhibits CrtN; reduces staphyloxanthin, enhances antibiotic susceptibility	Synergizes with ampicillin/cefotaxime; reduces biofilm	Antifungal repurposing may have off-target effects
**Theaflavin 3,3'-digallate**	50 μg/mL inhibits Hla; preserves skin barrier	Anti-inflammatory, no resistance risk	Not specified
**Thymol-isatin hybrid**	MIC: 1.9 μM; inhibits biofilm and staphyloxanthin	Low toxicity in *Galleria mellonella*; modulates immune response	Early-stage research
**Tilmicosin**	Reduced biofilm formation; synergistic with oxacillin.	Macrolide repurposing; targets mature biofilms.	Potential off-target effects due to antibiotic class.
**Trehalose-functionalized AuNPs**	Reduces bacterial binding to HUVECs	Non-toxic; targets bacterial-specific metabolism	Limited to in vitro models
**Triterpenoid acids**	Inhibits agr system; reduces dermonecrotic lesions (murine model)	Targets quorum sensing; no resistance pressure	Not specified
**Truncated SEA aptamers**	Enhanced binding specificity (lower Kd) for SEA detection	Reduced production time/cost compared to full-length aptamers	Requires validation in complex matrices (e.g., food)
**TST1N-224 (VraRC inhibitor)**	Restored vancomycin/methicillin susceptibility in VISA (IC50 = 60.2 μM).	Adjuvant potential with antibiotics.	Moderate binding affinity (KD = 23.4 μM).
**Vaccenic Acid**	Inhibits biofilm (40–60%) and virulence factors at sub-MIC.	Broad-spectrum (MRSA and *C. violaceum*); targets QS genes.	Limited *in vivo* data; moderate efficacy.
**Vancomycin-grafted Implants**	20-fold reduction in bacterial attachment *in vivo*	Long-term prevention of colonization; mitigates systemic inflammation	Residual bacteria persist on bone surfaces
**Vc-EAF**	MIC: 625 μg/mL (standard strains); synergistic with ampicillin	Natural origin; disrupts membrane permeability	High MIC (>2500 μg/mL) for some MRSA strains

**Verbascoside**	Inhibits SrtA-mediated adhesion; protects *Galleria* and murine models	Targets virulence without bactericidal pressure; strong SrtA binding affinity	Narrow focus on SrtA pathway
**Verteporfin**	Reduced bacterial load in murine wound infection; enhanced PMN killing.	Repurposed drug; targets redox sensing.	Limited data on long-term efficacy.
**Visomitin**	Bactericidal at >MIC; QS inhibition at sub-MIC; reduces hemolysin, biofilm.	Dual action (bactericidal + anti-QS); targets Agr system.	Higher concentrations needed for bactericidal effect.
**Xanthoangelol B, PM-56 (SaeR inhibitors)**	Suppressed hemolysins; improved survival in *Galleria mellonella* models.	Direct binding to SaeS; no growth inhibition.	Limited *in vivo* mammalian data.
**YycFG antisense RNA**	Reduced biofilm formation; increased cefoxitin susceptibility.	Enhances antibiotic efficacy; targets chronic infections.	Delivery challenges for antisense RNA *in vivo*.
**Zinc sulfate**	Synergy with antibiotics; downregulates *icaA, icaD, fnbA*	Enhances antibiotic efficacy, combats resistant strains	Not specified

Legend

AgNPs: Silver Nanoparticles; AgrA: Accessory gene regulator A; AI-2: Autoinducer-2; ARDS: Acute Respiratory Distress Syndrome; AuNPs: Gold Nanoparticles; *BfmR*: Biofilm regulatory protein; CFU: Colony Forming Units; ClfA/B: Clumping factors A and B; ClpP: Caseinolytic protease P; Coa: Coagulase; CrtM/N: Carotenoid biosynthesis enzymes; ΔGbind: Gibbs free energy change of binding; DNase: Deoxyribonuclease; eDNA: Extracellular DNA; EC50: Half Maximal Effective Concentration; EGCG: Epigallocatechin gallate; EPS: Extracellular Polymeric Substances; FIC: Fractional Inhibitory Concentration; FeoB: Ferrous iron transporter B; Fmoc-F: Fluorenylmethyloxycarbonyl-phenylalanine; GPX4: Glutathione Peroxidase 4; GrPE: Persimmon extract; Hla: Alpha-hemolysin; HPC: Hydroxypropyl Cellulose; HUVECs: Human Umbilical Vein Endothelial Cells; IC50: Half Maximal Inhibitory Concentration; ICA: Intercellular adhesion (*icaA/icaD* genes encode biofilm components); IL-1β/IL-6/IL-8: Interleukin-1 beta, Interleukin-6, Interleukin-8; LTA: Lipoteichoic Acid; LukAB: Leukocidin AB; MBIC: Minimum Biofilm Inhibitory Concentration; MDA: Malondialdehyde; MHC-II: Major Histocompatibility Complex class II; MIC: Minimum Inhibitory Concentration; MSCRAMM: Microbial Surface Components Recognizing Adhesive Matrix Molecules; MRSA: Methicillin-Resistant *S. aureus*; mAb: Monoclonal antibody; MD simulations: Molecular Dynamics simulations; NF-κB: Nuclear Factor kappa-light-chain-enhancer of activated B cells; NIR: Near-Infrared; PIA: Polysaccharide Intercellular Adhesin; PMN: Polymorphonuclear leukocytes; PoPE: Pomegranate extract; PSMs: Phenol-soluble modulins; PTT: Photothermal Therapy; PVL: Panton-Valentine Leukocidin; QS: Quorum Sensing; QSI: Quorum Sensing Inhibition; RNAIII: RNA molecule regulating virulence in *S. aureus*; ROS: Reactive Oxygen

Species; SarA: Staphylococcal accessory regulator A; SaeRS: Two-component system regulating virulence in *S. aureus*; scFv: Single-chain variable fragment (antibody fragment); SEA/SED/SEB: Staphylococcal Enterotoxins A, D, B; STX: Staphylococcal Toxin; SrtA/B: Sortase A/B; TCR: T-Cell Receptor; THC: Delta-9-Tetrahydrocannabinol; TNF-α: Tumor Necrosis Factor alpha; TSST-1: Toxic Shock Syndrome Toxin-1; VBNC: Viable But Non-Culturable; VISA: Vancomycin-Intermediate *S. aureus*; VRE: Vancomycin-Resistant Enterococci; VraS/R: Two-component system involved in cell wall stress response; vWbp: von Willebrand factor-binding protein.
